# Author Correction: Functional dissection of inherited non-coding variation influencing multiple myeloma risk

**DOI:** 10.1038/s41467-022-35411-1

**Published:** 2022-12-13

**Authors:** Ram Ajore, Abhishek Niroula, Maroulio Pertesi, Caterina Cafaro, Malte Thodberg, Molly Went, Erik L. Bao, Laura Duran-Lozano, Aitzkoa Lopez de Lapuente Portilla, Thorunn Olafsdottir, Nerea Ugidos-Damboriena, Olafur Magnusson, Mehmet Samur, Caleb A. Lareau, Gisli H. Halldorsson, Gudmar Thorleifsson, Gudmundur L. Norddahl, Kristbjorg Gunnarsdottir, Asta Försti, Hartmut Goldschmidt, Kari Hemminki, Frits van Rhee, Scott Kimber, Adam S. Sperling, Martin Kaiser, Kenneth Anderson, Ingileif Jonsdottir, Nikhil Munshi, Thorunn Rafnar, Anders Waage, Niels Weinhold, Unnur Thorsteinsdottir, Vijay G. Sankaran, Kari Stefansson, Richard Houlston, Björn Nilsson

**Affiliations:** 1 Hematology and Transfusion Medicine, Department of Laboratory Medicine, BMC B13, 221 84 Lund, Sweden; 2 Broad Institute of Massachusetts Institute of Technology and Harvard University, 415 Main Street, Boston, MA 02142 USA; 3 Division of Genetics and Epidemiology, The Institute of Cancer Research, 123 Old Brompton Road, London, SW7 3RP United Kingdom; 4 Division of Hematology/Oncology, Boston Children’s Hospital, Harvard Medical School, Boston, MA USA; 5 Dana-Farber Cancer Institute, Harvard Medical School, Boston, MA USA; 6 deCODE Genetics/Amgen Inc., Sturlugata 8, 101, Reykjavik, Iceland; 7 German Cancer Research Center (DKFZ), Im Neuenheimer Feld 580, D-69120 Heidelberg, Germany; 8 Hopp Children’s Cancer Center, Heidelberg, Germany; 9 Department of Internal Medicine V, University Hospital of Heidelberg, 69120 Heidelberg, Germany; 10 Faculty of Medicine and Biomedical Center in Pilsen, Charles University in Prague, Prague, 30605 Czech Republic; 11 Department of Cancer Research and Molecular Medicine, Norwegian University of Science and Technology, Box 8905, N-7491 Trondheim, Norway; 12 Harvard Stem Cell Institute, Cambridge, MA USA

**Keywords:** Cancer epigenetics, Cancer genomics, Quantitative trait loci, Myeloma, Genetics research

Correction to: *Nature Communications* 10.1038/s41467-021-27666-x, published online 10 January 2022

In this article the grant number 847583 relating to the European Research Council for Björn Nilsson was omitted in the Acknowledgements section. The original article has been corrected.

