# Supplementary material for: Functional dissection of inherited non-coding variation influencing multiple myeloma risk
Source: Nat Commun. 2022 Jan 10;13:151. doi: 10.1038/s41467-021-27666-x (PMC8748989; doi:10.1038/s41467-021-27666-x)
Supplement: Supplementary file 1 — Supplementary Information [file 41467_2021_27666_MOESM1_ESM.pdf]

Supplementary Figure 1

g-chromVAR *P*-values for enrichment of MM risk variants within genomic regions of accessible chromatin in blood cell populations, weighted by posterior probabilities for each variant. Abbreviations: B-cell (B), common lymphoid progenitor (CLP), common myeloid progenitor (CMP), erythroid precursors (ERY), granulocyte-monocyte progenitor (GMP), granulocyte (GRAN), hematopoietic stem cell (HSC), lymphoid-primed multipotent progenitors (LMPP), myeloid dendritic cell (mDC), megakaryocyte (MEGA), megakaryocyte-erythrocyte progenitor (MEP), monocyte (MONO), multipotent progenitor (MPP), natural killer cells (NK), plasma cell (PC), plasmacytoid dendritic cell (pDC), CD4+ T-cells (CD4), CD8+ T-cells (CD8).

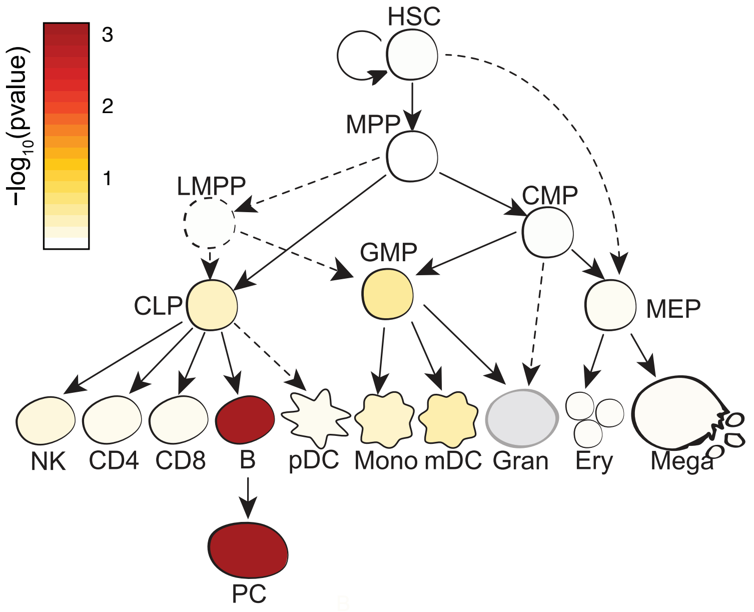

Supplementary Figure 2

Enrichment of genes at MM risk loci across hematopoietic cell types. **(a)** Using gene expression profiles of sorted blood cell populations (PMID 21241896, 21994415, 20574050, 21430775 and 17023574; **Online Methods**), we looked for enrichment of expression of genes located in MM-associated regions. We identified B-cells and plasma cells from normal donors and patients with MM, smoldering MM or monoclonal gammopathy of unknown significance (MGUS; the precursor disease of MM) as the most enriched cell types. **(b)** Similar results were obtained with an independent set of RNA-sequencing data (PMID 30858613). Abbreviations: B-cells (BCELL), basophil (BASO), common lymphoid progenitor (CLP), common myeloid progenitor (CMP), dendritic cell (DEND), eosinophil (EOS), erythroid precursors (ERY), granulocyte-monocyte progenitor (GMP), granulocyte (GRAN), hematopoietic stem cell (HSC), lymphoid-primed multipotent progenitors (LMPP), megakaryocyte (MEGA), megakaryocyte-erythrocyte progenitor (MEP), monoclonal gammopathy of unknown significance (MGUS), monocyte (MONO), multiple myeloma (MM), multipotent progenitor (MPP), natural killer cells (NK, NKa), plasma cells (PC), pre-B-cell (PRE BCELL), CD4+ T-cells (TCELL CD4), CD8+ T-cells (TCELL CD8).

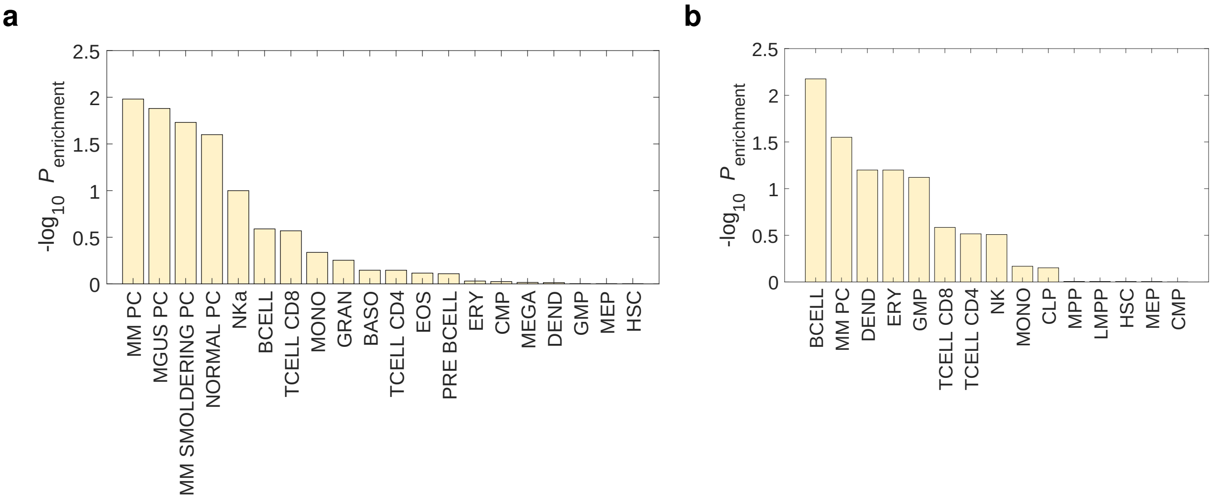

Supplementary Figure 3

Quantile-quantile plots of *P*-values for association between lead variants at MM risk loci and blood IgA, IgG, and IgM levels across 16,883 Icelanders. Lead variants showing Bonferroni-significant association are labelled with the names of the main candidate genes at their respective loci.

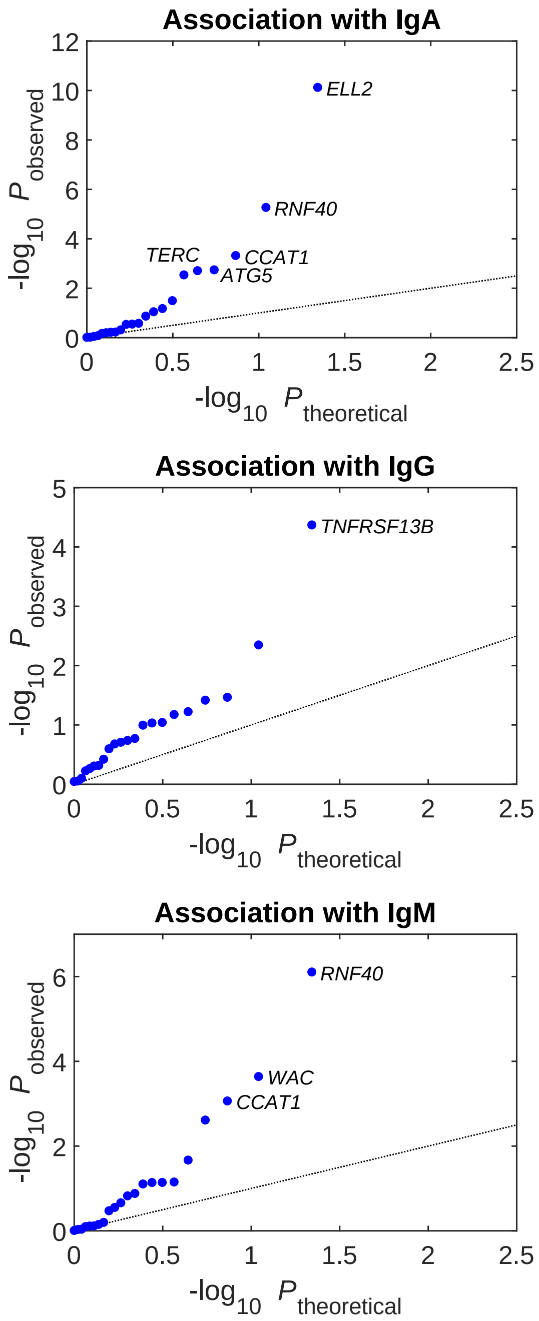

Supplementary Figure 4

We performed luciferase analyses for 20 variants in L363 cells, including 18 that were significant in both cell lines and the top-ranking variants at *RFWD3* and *CCDC71L*. All except rs3827644 showed effects in the same direction as in the MPRA. Left- and right-hand data represent the reference and alternative alleles, respectively. The y-axis indicates luciferase signal ($\log_2$-transformed, median-centered luciferase/renilla ratio). The bottom, middle and top of each box plot indicate the 25:th, 50:th, and 75:th percentiles. Whiskers indicate non-outlier minimum and maximum (1.5 $\times$ interquartile range from the bottom and top of the box). P-values for Student's t-test.

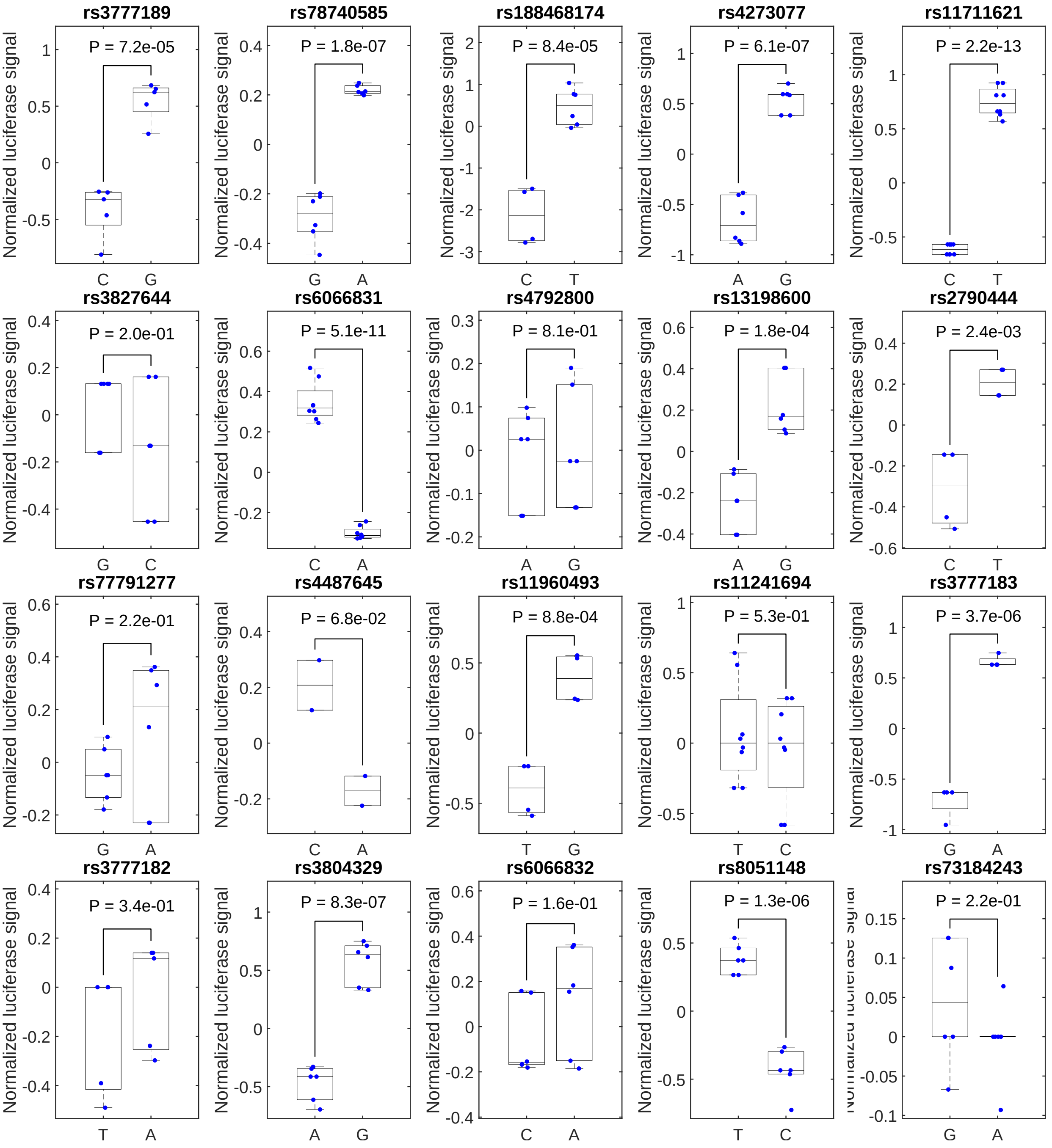

Supplementary Figure 5

Overarching analysis of variant statistics for the two MPRA screens. **(a,b):** g-chromVAR analysis showing enrichments of strong $\log_2$ scores within genomic regions with accessible chromatin in different blood cell types. In both screens, we detected an enrichment in regions with accessible chromatin in plasma cells, though the enrichment was most pronounced in L363. **c,d:** Distributions of variant P-values, with the estimated null distribution indicated in red. As shown, we observed a strong enrichment of low P-values in both cell lines. The proportion of variants following the null hypothesis (π_0; estimated here as two times the number of P-values above 0.5 divided by the total number of P values) was 56% for L363 and 76% for MOLP8. The lower π_0 in L363 is consistent with a higher signal-to-noise ratio and the stronger gChromVar signal for plasma cells.

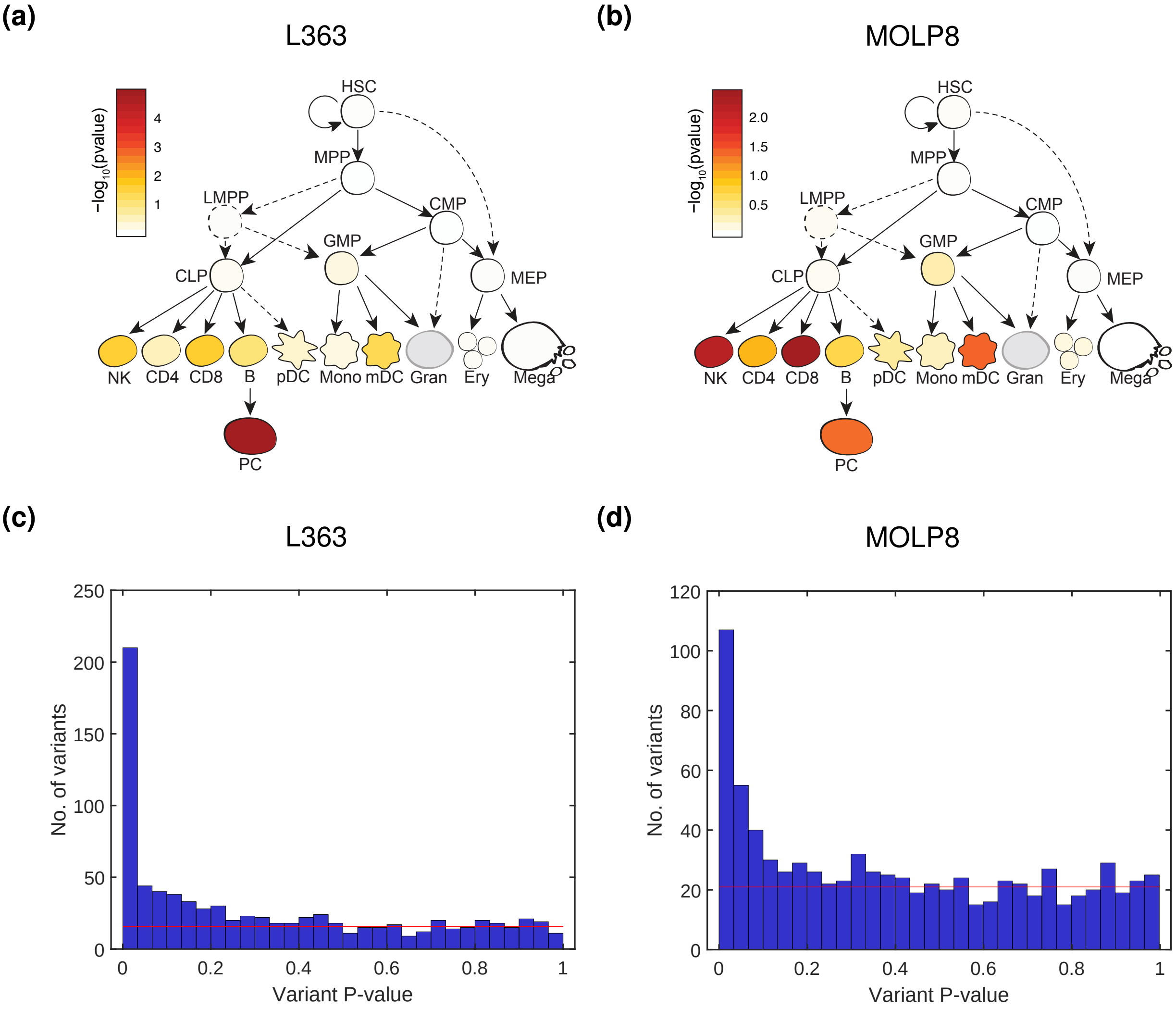

Supplementary Figure 6

Individual barcode activity estimates for the variants in **Table 1**. Data grouped by allele (reference allele to the left; alternative to the right), DNA strand (+ or -) and sliding window (variant at -20, 0 or +20 bp from the center of the 120-bp oligonucleotide representing the genomic context). Blue dots represent individual barcode activity estimates. The bottom, middle and top of each box plot represent the 25:th, 50:th, and 75:th percentiles. The whiskers represent the non-outlier minimum and maximum values, located at 1.5 times the interquartile range from the bottom and top of the box, respectively. The numbers above the brackets are *P*-values for two-sided Student's t-test. The final MPRAscore *P*-values, integrating all six genomic contexts, are given in **Table 1**.

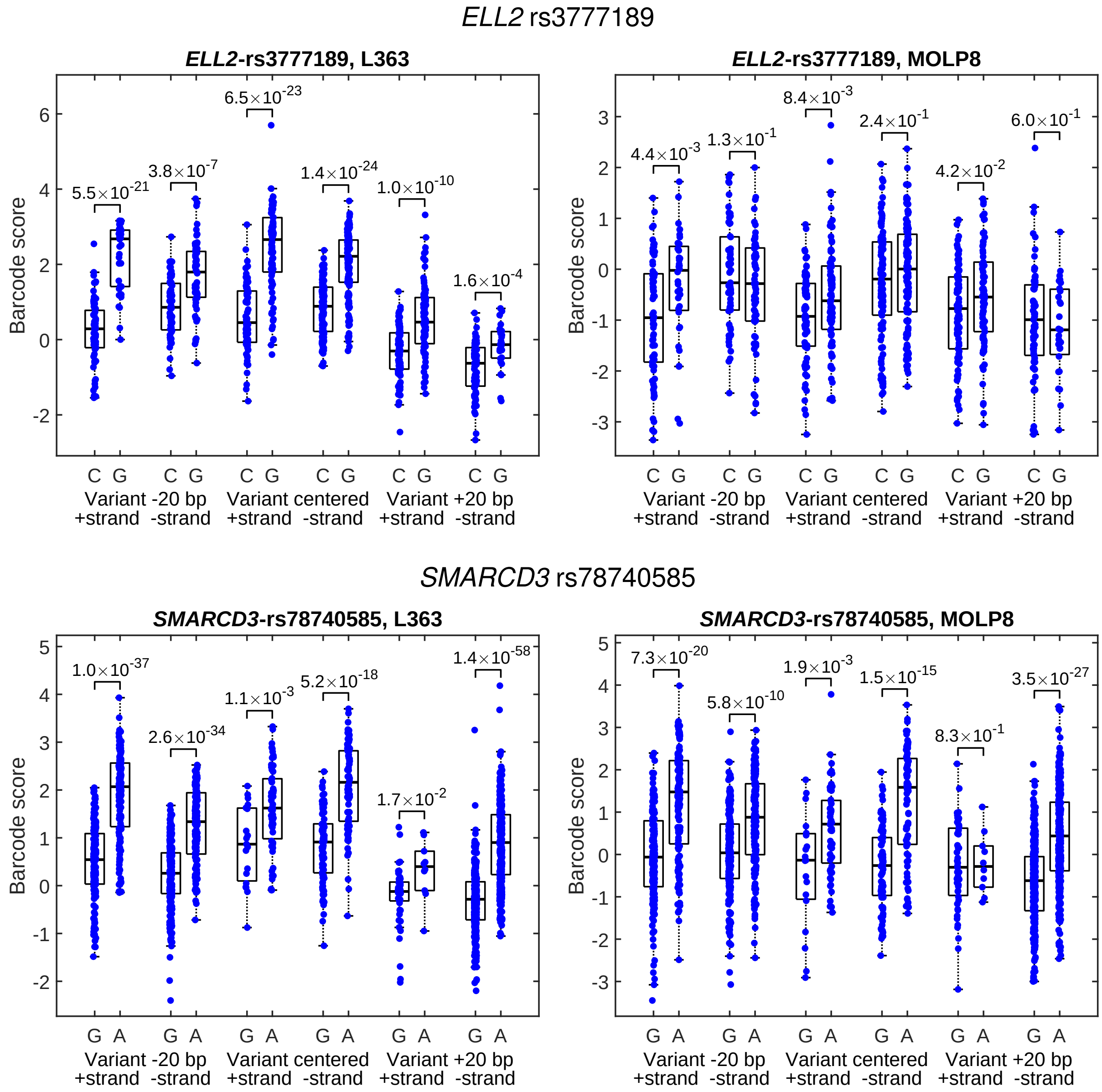

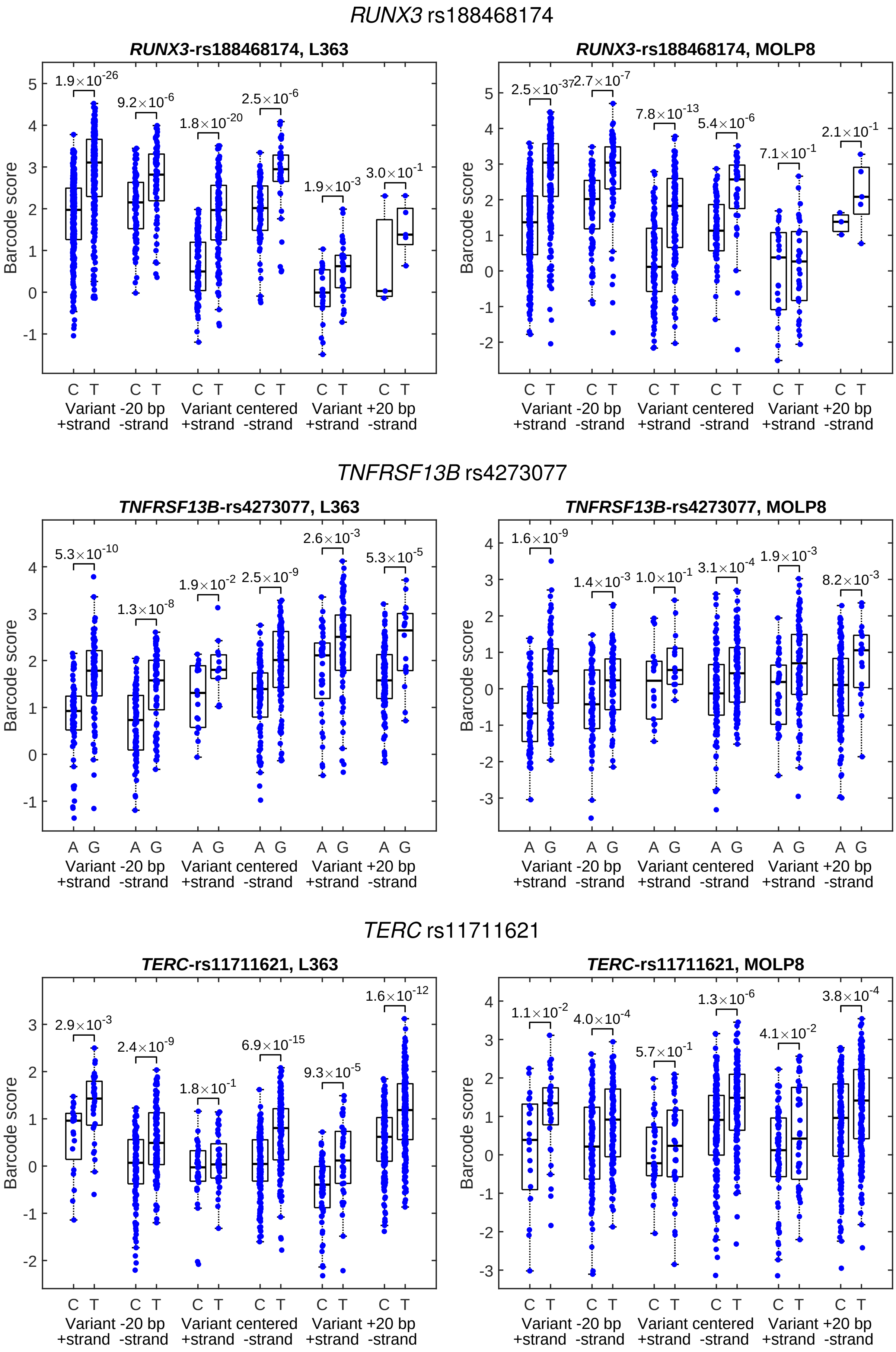

RUNX3 rs188468174
RUNX3-rs188468174, L363
1.9×10-26
9.2×10-6
1.8×10-20
2.5×10-6
1.9×10-3
3.0×10-1
Barcode score
C T C T C T C T C T C T
Variant -20 bp
Variant centered
Variant +20 bp
+strand -strand +strand -strand +strand -strand
RUNX3-rs188468174, MOLP8
2.5×10-37
2.7×10-7
7.8×10-13
5.4×10-6
7.1×10-1
2.1×10-1
Barcode score
C T C T C T C T C T C T
Variant -20 bp
Variant centered
Variant +20 bp
+strand -strand +strand -strand +strand -strand
TNFRSF13B rs4273077
TNFRSF13B-rs4273077, L363
5.3×10-10
1.3×10-8
1.9×10-2
2.5×10-9
2.6×10-3
5.3×10-5
Barcode score
A G A G A G A G A G A G
Variant -20 bp
Variant centered
Variant +20 bp
+strand -strand +strand -strand +strand -strand
TNFRSF13B-rs4273077, MOLP8
1.6×10-9
1.4×10-3
1.0×10-1
3.1×10-4
1.9×10-3
8.2×10-3
Barcode score
A G A G A G A G A G A G
Variant -20 bp
Variant centered
Variant +20 bp
+strand -strand +strand -strand +strand -strand
TERC rs11711621
TERC-rs11711621, L363
2.9×10-3
2.4×10-9
1.8×10-1
6.9×10-15
9.3×10-5
1.6×10-12
Barcode score
C T C T C T C T C T C T
Variant -20 bp
Variant centered
Variant +20 bp
+strand -strand +strand -strand +strand -strand
TERC-rs11711621, MOLP8
1.1×10-2
4.0×10-4
5.7×10-1
1.3×10-6
4.1×10-2
3.8×10-4
Barcode score
C T C T C T C T C T C T
Variant -20 bp
Variant centered
Variant +20 bp
+strand -strand +strand -strand +strand -strand

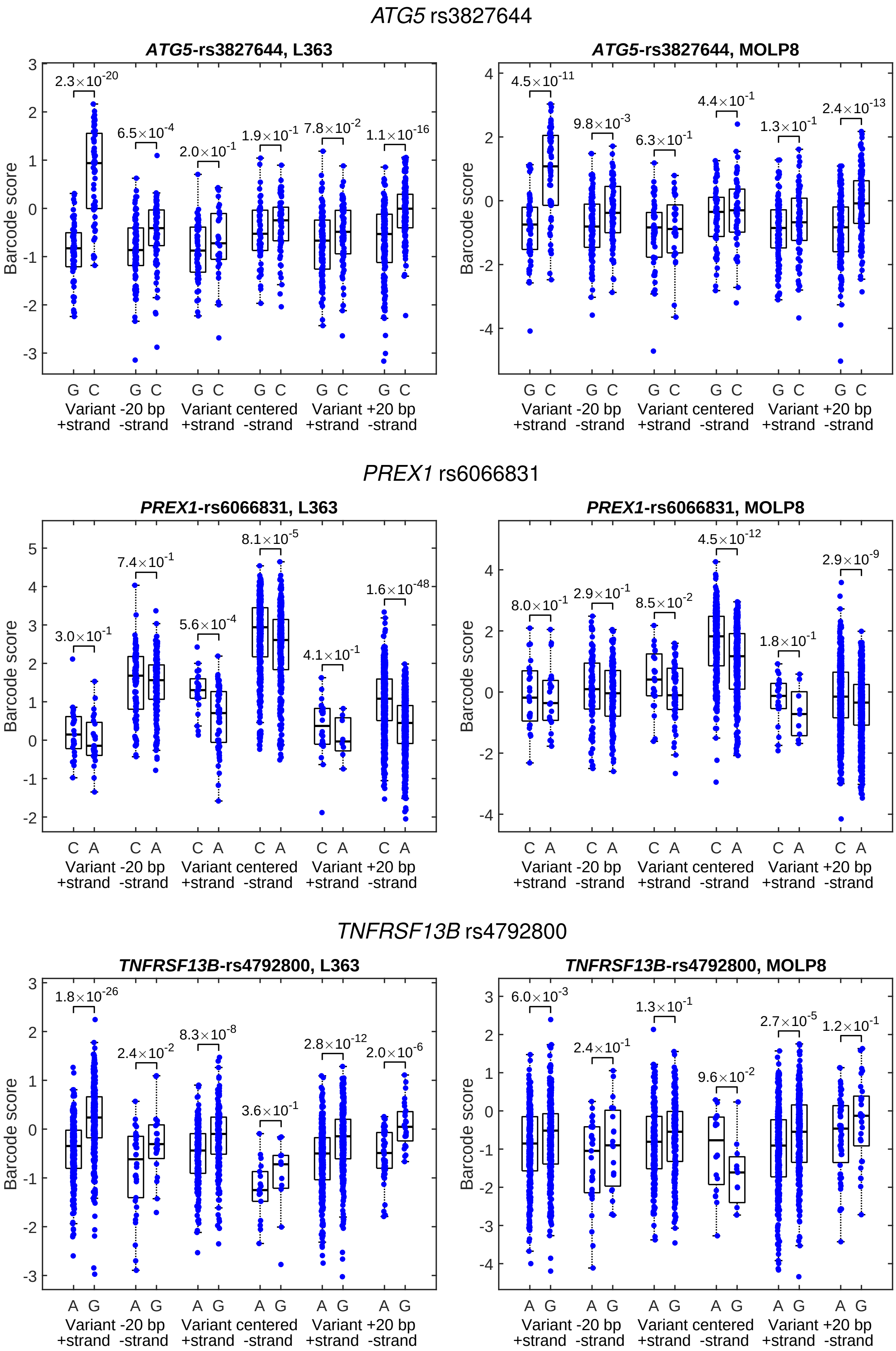

ATG5 rs3827644
ATG5-rs3827644, L363
2.3×10-20
6.5×10-4
2.0×10-1
1.9×10-1
7.8×10-2
1.1×10-16
Barcode score
G C
Variant -20 bp
Variant centered
Variant +20 bp
+strand -strand
ATG5-rs3827644, MOLP8
4.5×10-11
9.8×10-3
6.3×10-1
4.4×10-1
1.3×10-1
2.4×10-13
PREX1 rs6066831
PREX1-rs6066831, L363
3.0×10-1
7.4×10-1
5.6×10-4
8.1×10-5
4.1×10-1
1.6×10-48
C A
PREX1-rs6066831, MOLP8
8.0×10-1
2.9×10-1
8.5×10-2
4.5×10-12
1.8×10-1
2.9×10-9
TNFRSF13B rs4792800
TNFRSF13B-rs4792800, L363
1.8×10-26
2.4×10-2
8.3×10-8
3.6×10-1
2.8×10-12
2.0×10-6
A G
TNFRSF13B-rs4792800, MOLP8
6.0×10-3
2.4×10-1
1.3×10-1
9.6×10-2
2.7×10-5
1.2×10-1

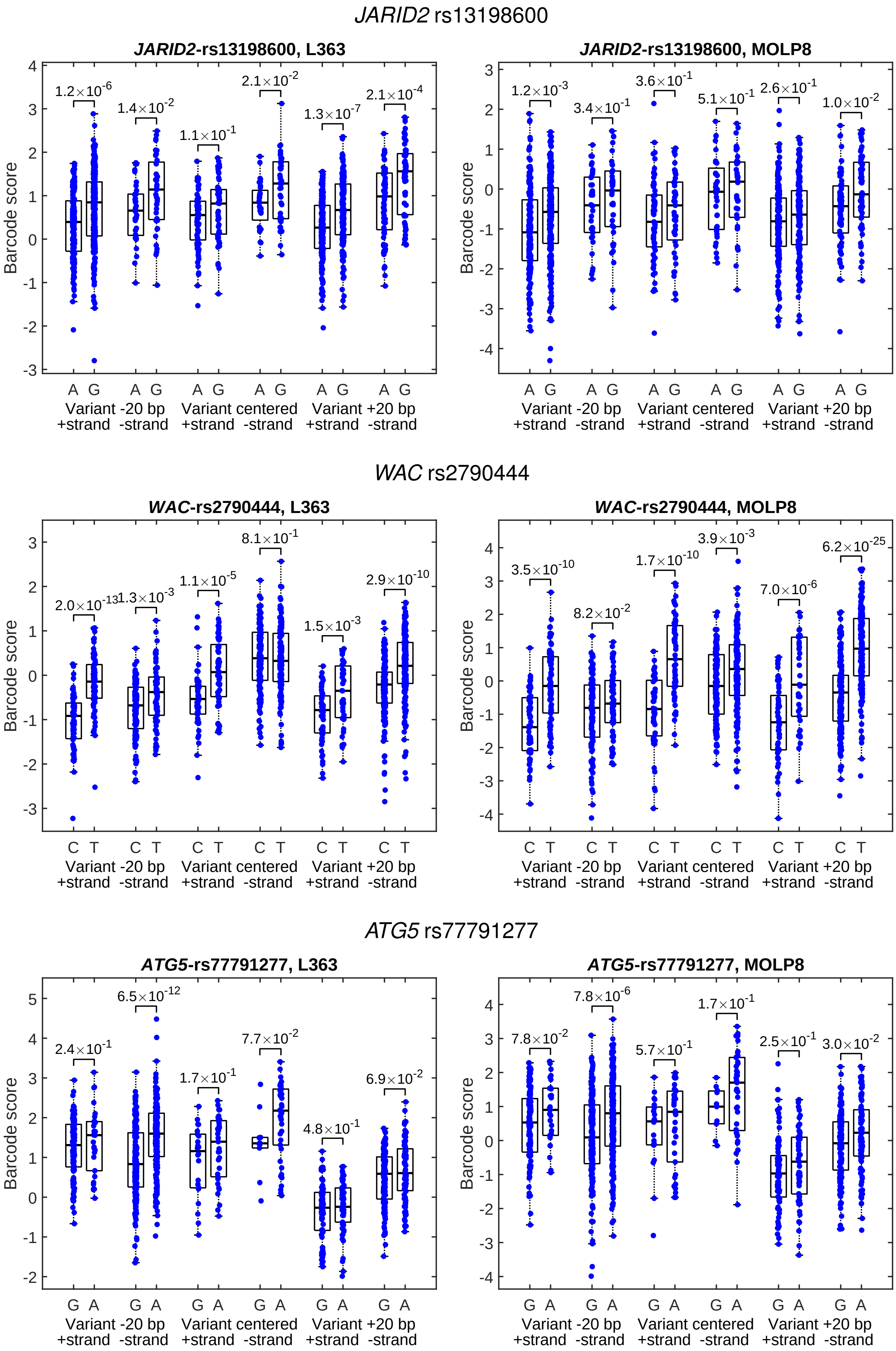
JARID2 rs13198600
JARID2-rs13198600, L363
1.2×10^{-6} 1.4×10^{-2} 1.1×10^{-1} 2.1×10^{-2} 1.3×10^{-7} 2.1×10^{-4}
Barcode score
A G A G A G A G A G A G
Variant -20 bp +strand -strand
Variant centered +strand -strand
Variant +20 bp +strand -strand
JARID2-rs13198600, MOLP8
1.2×10^{-3} 3.4×10^{-1} 3.6×10^{-1} 5.1×10^{-1} 2.6×10^{-1} 1.0×10^{-2}
WAC rs2790444
WAC-rs2790444, L363
2.0×10^{-13} 1.3×10^{-3} 1.1×10^{-5} 8.1×10^{-1} 1.5×10^{-3} 2.9×10^{-10}
C T C T C T C T C T C T
WAC-rs2790444, MOLP8
3.5×10^{-10} 8.2×10^{-2} 1.7×10^{-10} 3.9×10^{-3} 7.0×10^{-6} 6.2×10^{-25}
ATG5 rs77791277
ATG5-rs77791277, L363
2.4×10^{-1} 6.5×10^{-12} 1.7×10^{-1} 7.7×10^{-2} 4.8×10^{-1} 6.9×10^{-2}
G A G A G A G A G A G A
ATG5-rs77791277, MOLP8
7.8×10^{-2} 7.8×10^{-6} 5.7×10^{-1} 1.7×10^{-1} 2.5×10^{-1} 3.0×10^{-2}

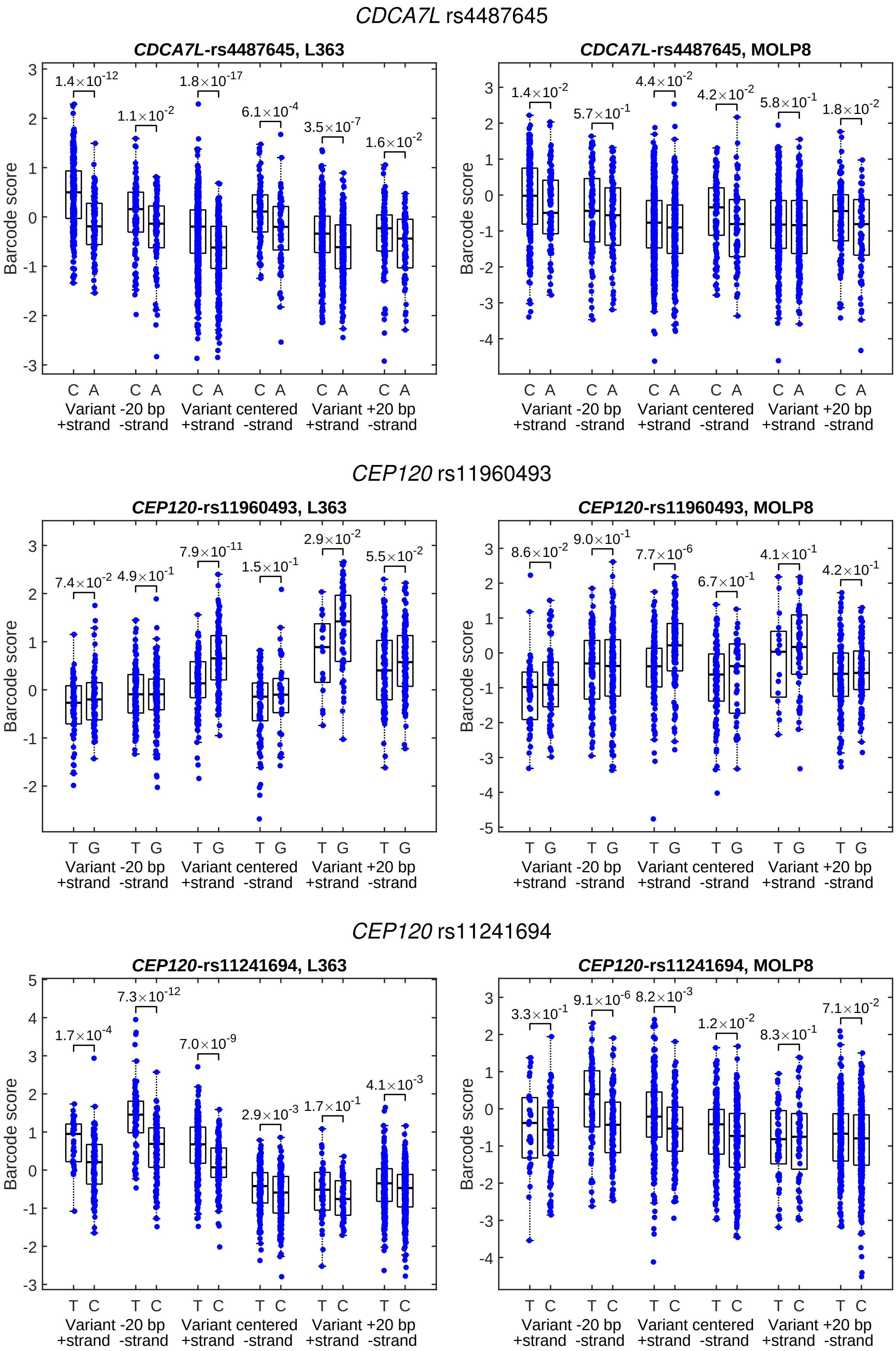
CDCA7L rs4487645
***CDCA7L*-rs4487645, L363**
1.4×10^{-12}
1.1×10^{-2}
1.8×10^{-17}
6.1×10^{-4}
3.5×10^{-7}
1.6×10^{-2}
Barcode score
C A C A C A C A C A C A
Variant -20 bp Variant centered Variant +20 bp
+strand -strand +strand -strand +strand -strand
***CDCA7L*-rs4487645, MOLP8**
1.4×10^{-2}
5.7×10^{-1}
4.4×10^{-2}
4.2×10^{-2}
5.8×10^{-1}
1.8×10^{-2}
Barcode score
C A C A C A C A C A C A
Variant -20 bp Variant centered Variant +20 bp
+strand -strand +strand -strand +strand -strand
CEP120 rs11960493
***CEP120*-rs11960493, L363**
7.4×10^{-2}
4.9×10^{-1}
7.9×10^{-11}
1.5×10^{-1}
2.9×10^{-2}
5.5×10^{-2}
Barcode score
T G T G T G T G T G T G
Variant -20 bp Variant centered Variant +20 bp
+strand -strand +strand -strand +strand -strand
***CEP120*-rs11960493, MOLP8**
8.6×10^{-2}
9.0×10^{-1}
7.7×10^{-6}
6.7×10^{-1}
4.1×10^{-1}
4.2×10^{-1}
Barcode score
T G T G T G T G T G T G
Variant -20 bp Variant centered Variant +20 bp
+strand -strand +strand -strand +strand -strand
CEP120 rs11241694
***CEP120*-rs11241694, L363**
1.7×10^{-4}
7.3×10^{-12}
7.0×10^{-9}
2.9×10^{-3}
1.7×10^{-1}
4.1×10^{-3}
Barcode score
T C T C T C T C T C T C
Variant -20 bp Variant centered Variant +20 bp
+strand -strand +strand -strand +strand -strand
***CEP120*-rs11241694, MOLP8**
3.3×10^{-1}
9.1×10^{-6}
8.2×10^{-3}
1.2×10^{-2}
8.3×10^{-1}
7.1×10^{-2}
Barcode score
T C T C T C T C T C T C
Variant -20 bp Variant centered Variant +20 bp
+strand -strand +strand -strand +strand -strand

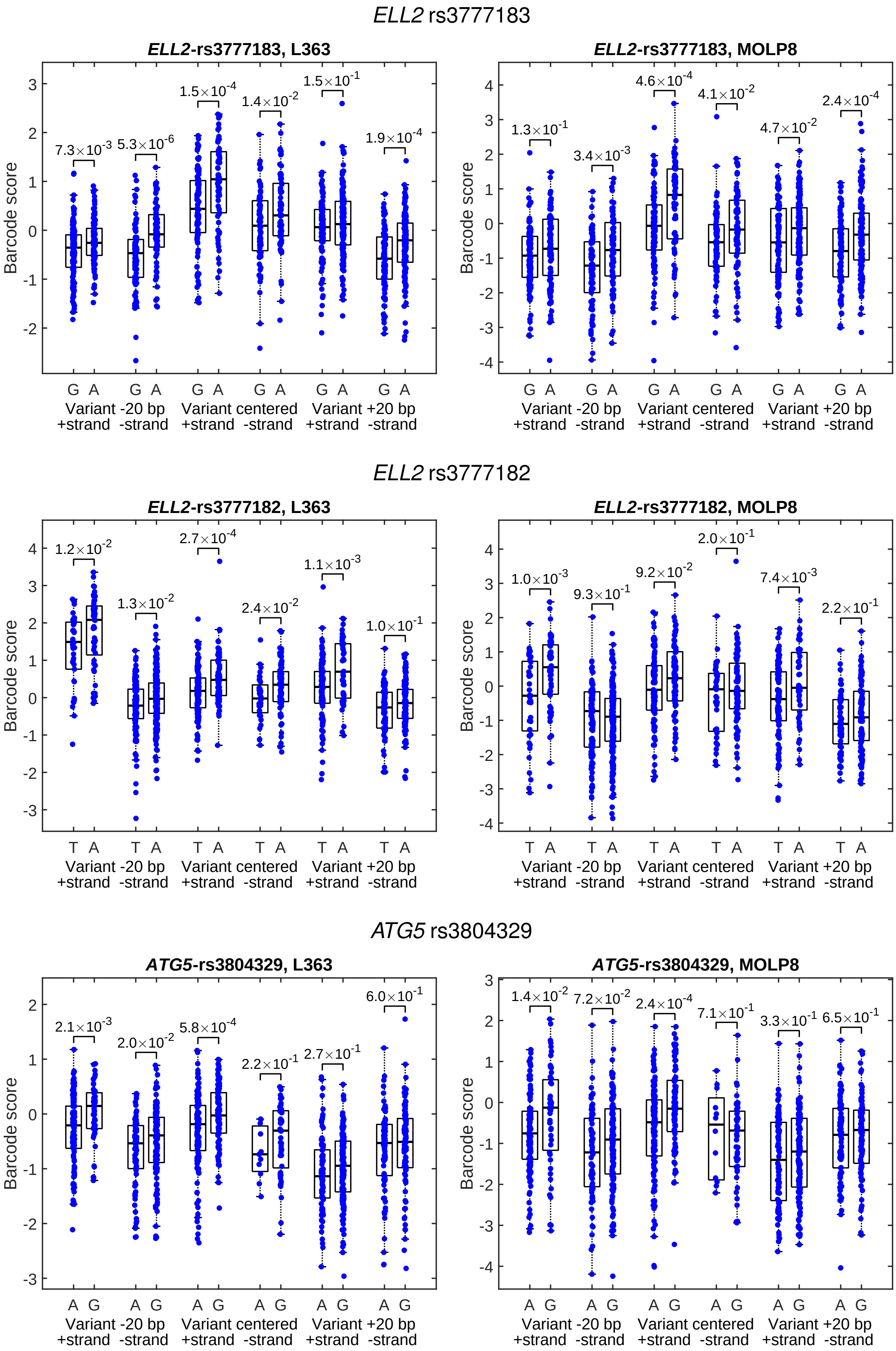
ELL2 rs3777183
ELL2-rs3777183, L363
ELL2-rs3777183, MOLP8
Barcode score
7.3×10-3
5.3×10-6
1.5×10-4
1.4×10-2
1.5×10-1
1.9×10-4
1.3×10-1
3.4×10-3
4.6×10-4
4.1×10-2
4.7×10-2
2.4×10-4
G A G A G A G A G A G A
Variant -20 bp
Variant centered
Variant +20 bp
+strand -strand +strand -strand +strand -strand
ELL2 rs3777182
ELL2-rs3777182, L363
ELL2-rs3777182, MOLP8
1.2×10-2
1.3×10-2
2.7×10-4
2.4×10-2
1.1×10-3
1.0×10-1
1.0×10-3
9.3×10-1
9.2×10-2
2.0×10-1
7.4×10-3
2.2×10-1
T A T A T A T A T A T A
ATG5 rs3804329
ATG5-rs3804329, L363
ATG5-rs3804329, MOLP8
2.1×10-3
2.0×10-2
5.8×10-4
2.2×10-1
2.7×10-1
6.0×10-1
1.4×10-2
7.2×10-2
2.4×10-4
7.1×10-1
3.3×10-1
6.5×10-1
A G A G A G A G A G A G

PREX1 rs6066832

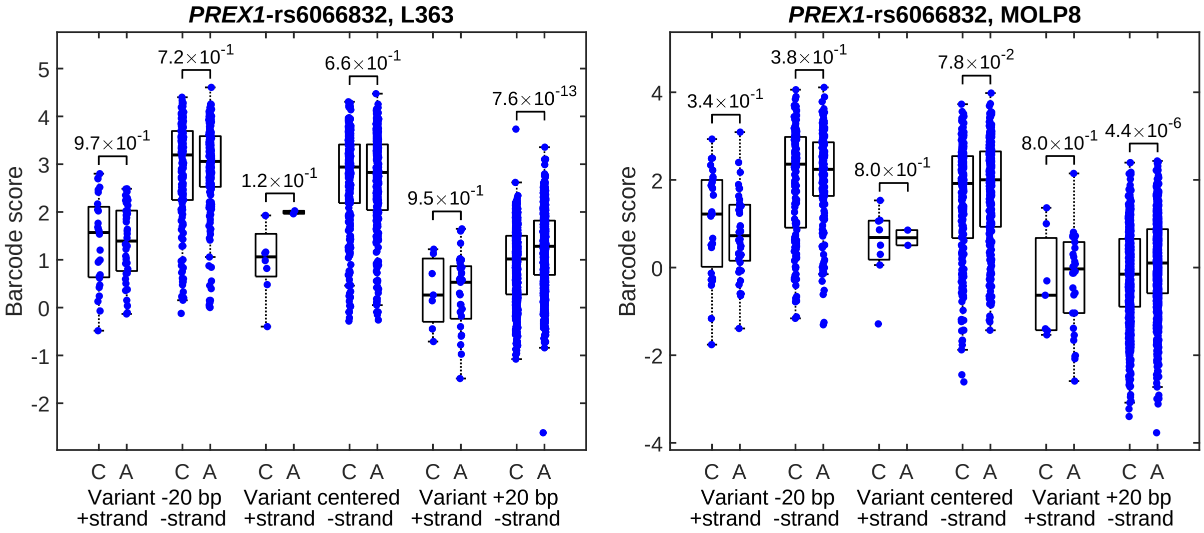

Supplementary Figure 7

Full gel scans for **Fig. 5**. **(a)** Full scan for electromobility shift assay showing selective binding of IRF4 to rs78740585-A probe (**Fig. 5c**). Arrow indicates supershift with antibody towards IRF4. Abbreviations: labeled probe (LP), L363 nuclear extract (NE), unlabeled probe (ULP), antibody against IRF4 (α-IRF4). **(b)** Full scan for Western blot for IRF4 (**Fig. 5e**), alongside colorimetric ladder (BioRad 161-0374). **(c)** Staining for ACTB to confirm equal loading.

(a)

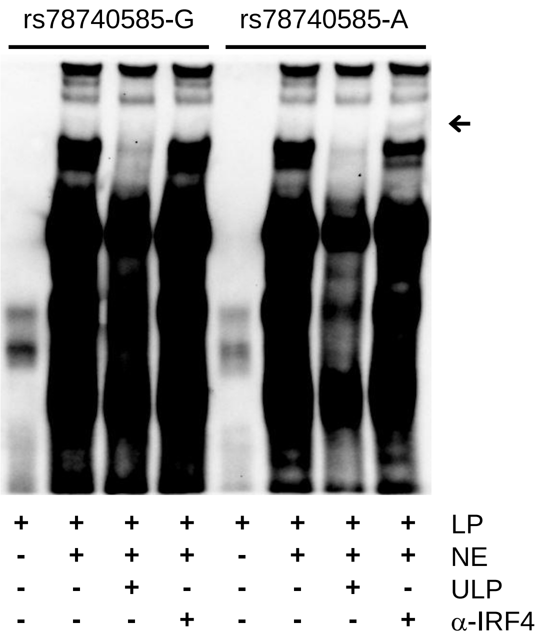

(b)

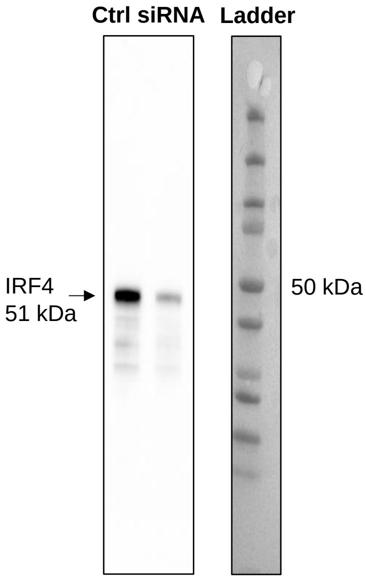

(c)

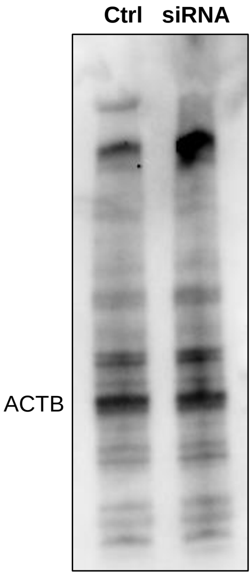

Supplementary Figure 8

Chromatin looping interactions at the selected loci, as determined by PCHi-C. The six panels show interactions with: **(a)** the *SMARCD3* promoter; **(b)** the *WAC* promoter; **(c)** the *ELL2* promoter; **(d)** the *CDCA7L* promoter; **(e)** the *PREX1* promoter; and **(f)** the *CEP120* promoter. The interactions shown are within 3 Mb from the promoter bait, and have $-\log_{10}$(CHiCAGO *P*-score)$\geq$2.

(a)

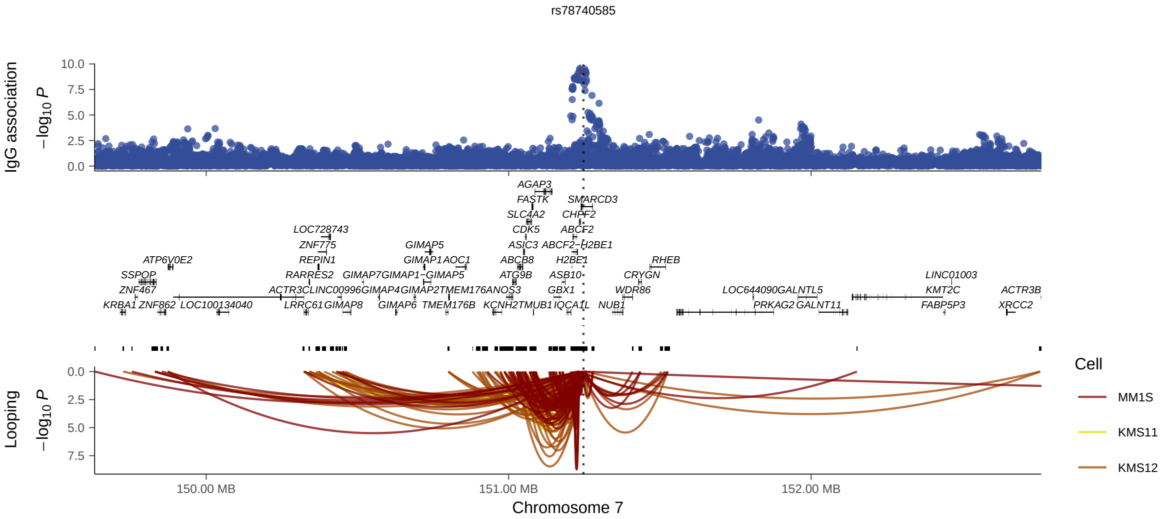

(b)

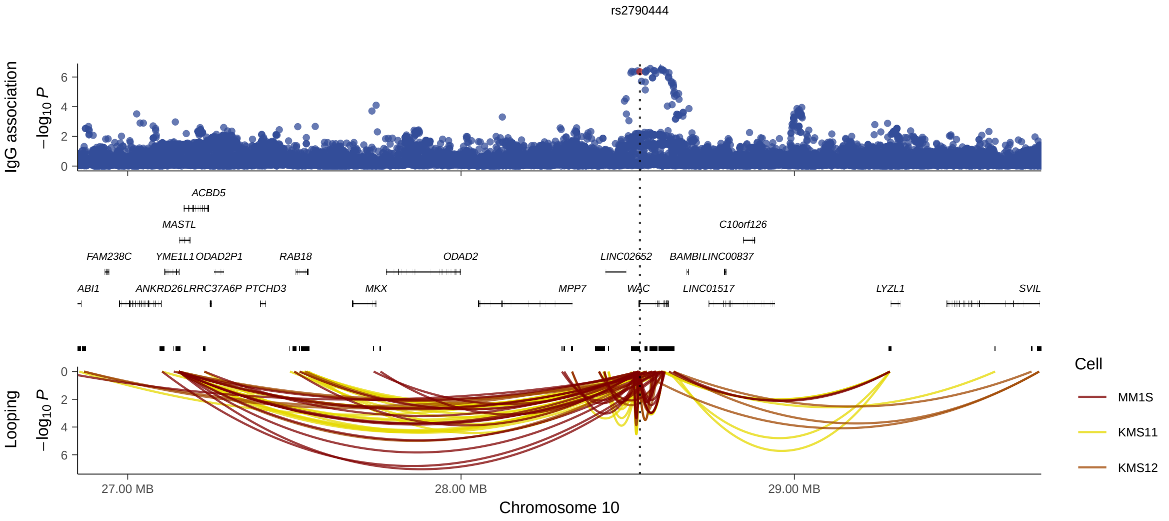

(c)

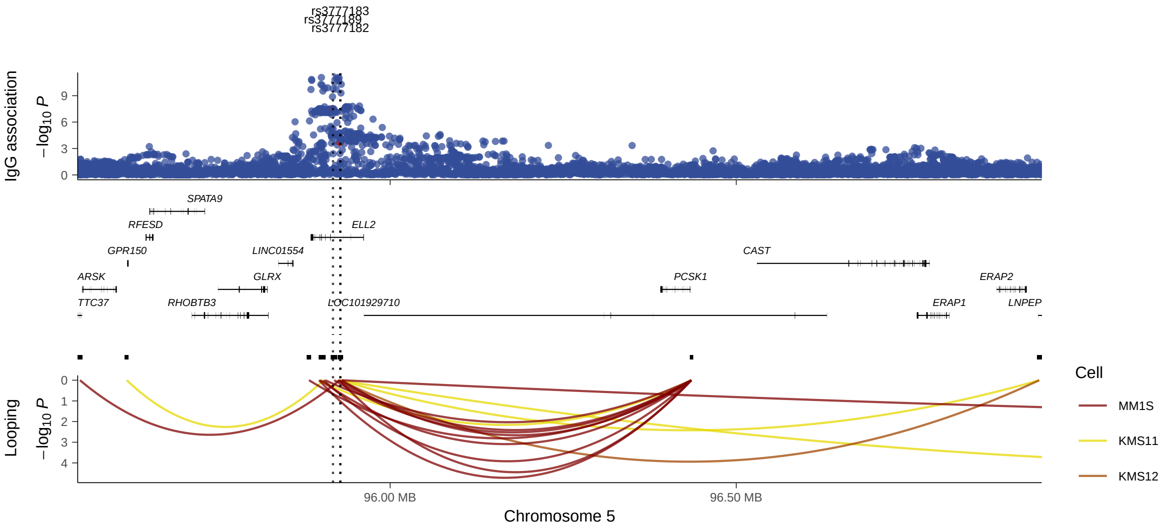
rs3777183
rs3777189
rs3777182
IgG association
−log10 P
9
6
3
0
SPATA9
RFESD
ELL2
GPR150
LINC01554
CAST
ARSK
GLRX
PCSK1
ERAP2
TTC37
RHOBTB3
LOC101929710
ERAP1
LNPEP
Looping
−log10 P
0
1
2
3
4
96.00 MB
96.50 MB
Chromosome 5
Cell
MM1S
KMS11
KMS12

(d)

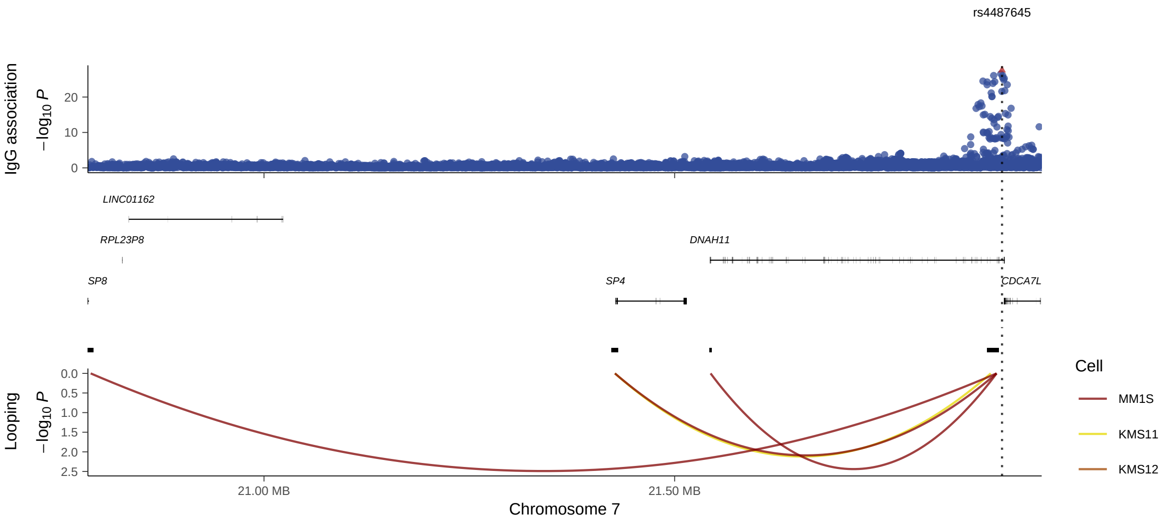
rs4487645
IgG association
−log10 P
20
10
0
LINC01162
RPL23P8
DNAH11
SP8
SP4
CDCA7L
Looping
−log10 P
0.0
0.5
1.0
1.5
2.0
2.5
21.00 MB
21.50 MB
Chromosome 7
Cell
MM1S
KMS11
KMS12

(e)

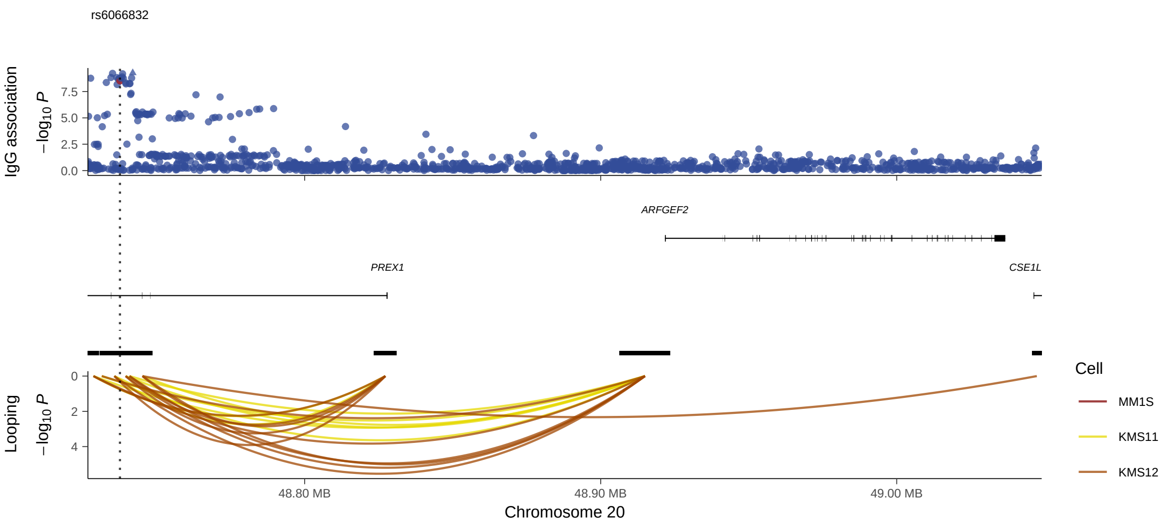
rs6066832
IgG association
−log10 P
7.5
5.0
2.5
0.0
ARFGEF2
PREX1
CSE1L
Looping
−log10 P
0
2
4
48.80 MB
48.90 MB
49.00 MB
Chromosome 20
Cell
MM1S
KMS11
KMS12

(f)

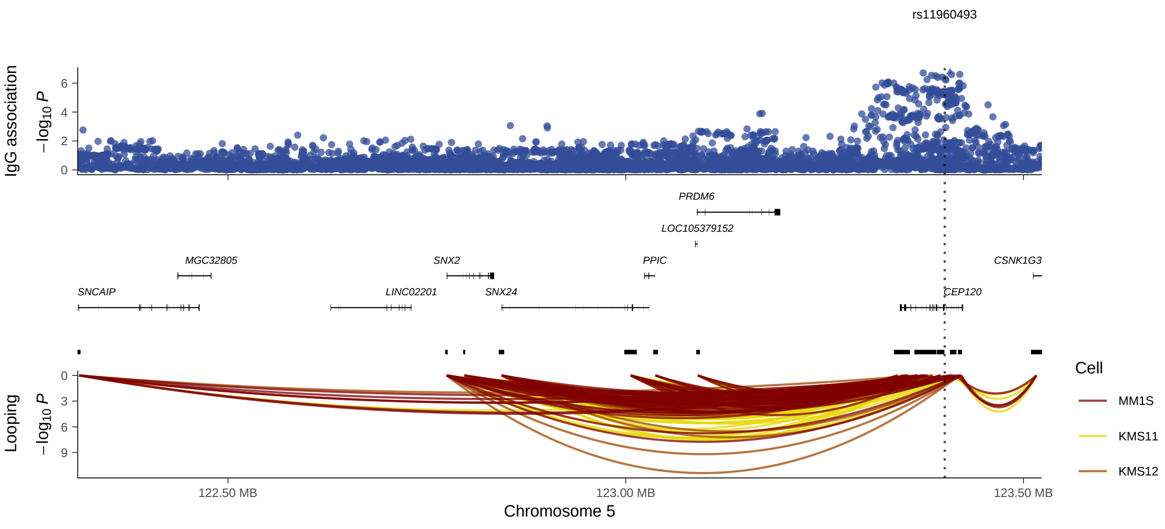
rs11960493
IgG association
−log10 P
6
4
2
0
PRDM6
LOC105379152
MGC32805
SNX2
PPIC
CSNK1G3
SNCAIP
LINC02201
SNX24
CEP120
Looping
−log10 P
0
3
6
9
122.50 MB
123.00 MB
123.50 MB
Chromosome 5
Cell
MM1S
KMS11
KMS12

Supplementary Figure 9

Full gel scans for **Fig. 6**. **(a)** Full scan for electromobility shift assay showing selective binding of POU2F1 to rs2790444-T probe. Arrow indicates supershift with antibody against POU2F1 (**Fig. 6c**). **(b)** Full scan for PCR confirming CRISPR deletion of the rs2790444 variant-harboring region (**Fig. 6f**). Abbreviations: labeled probe (LP), L363 nuclear extract (NE), unlabeled probe (ULP), antibody against POU2F1 (α-POU2F1). **(c)** Full scan for Western blot for POU2F1 (**Fig. 5e**), alongside colorimetric ladder (BioRad 161-0374). **(d)** Staining for ACTB to confirm equal loading.

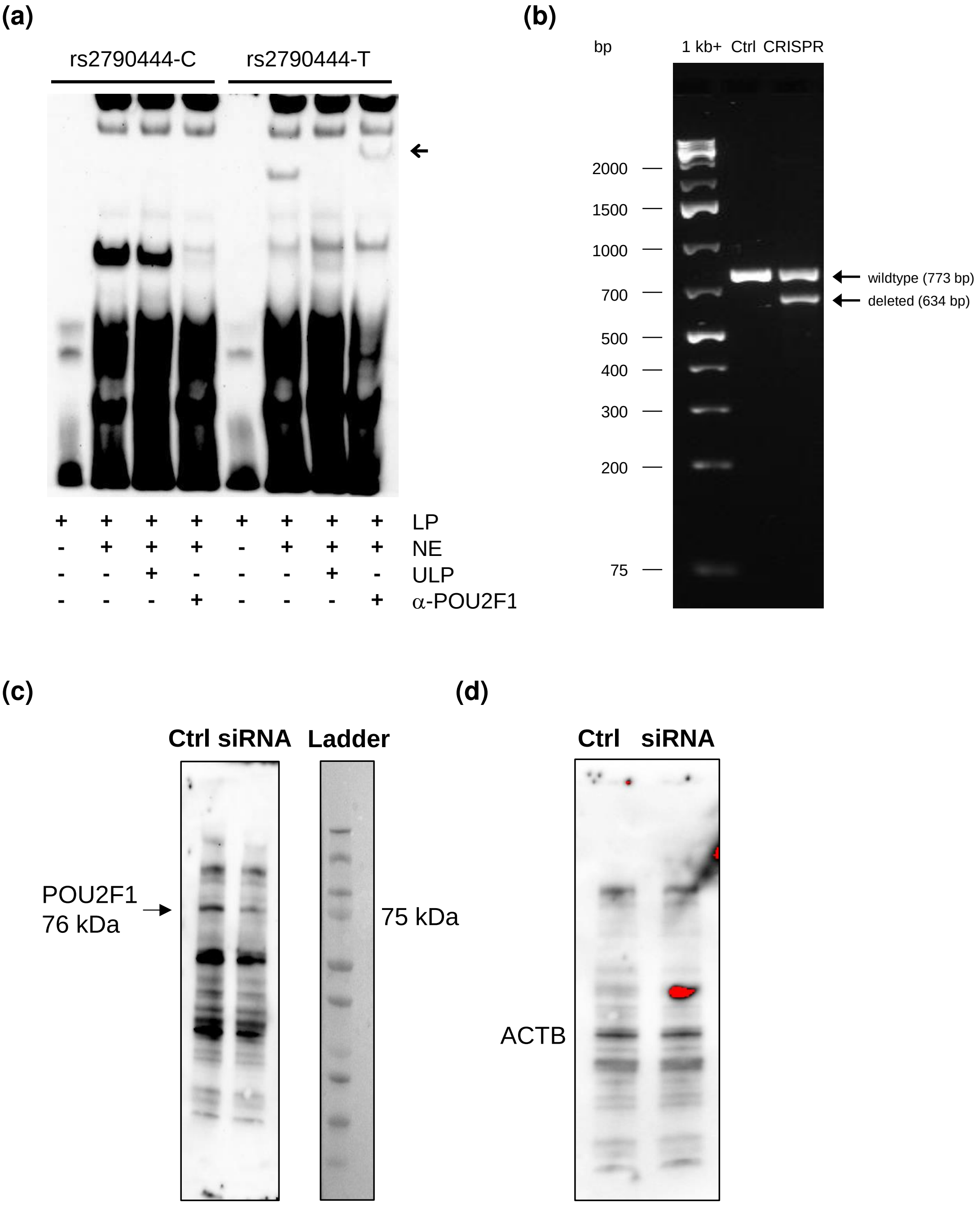

Supplementary Figure 10

Full gel scans of agarose gels for PCR to confirm CRISPR-deletion of variant-harboring regions in **Fig. 7**. **(a)** rs3777182-83 region. (**(b)** rs3777189 region **(c)** rs4487645 region.

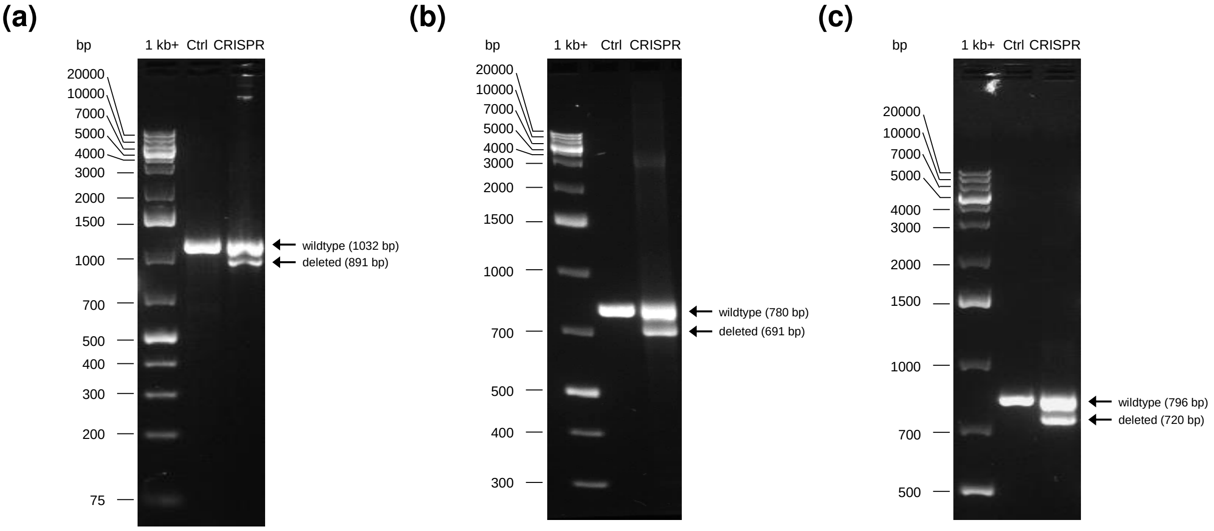

Supplementary Figure 11

Discovery caQTL analysis in 56 samples revealed regions with allele-dependent accessibility around rs4487645 and rs11960493, and borderline association at rs78740585. Average ATAC-seq signal for individuals with different genotypes indicated by yellow, orange and red lines. False discovery rate (-log$_{10}$ Q-value) for correlation between ATAC-seq signal intensity and lead variant genotype indicated in dashed blue. Regions called allele-dependent in light blue. Variants showing association with MM indicated by red circles (P_{MM} = P value for association as in **Fig. 1a**).

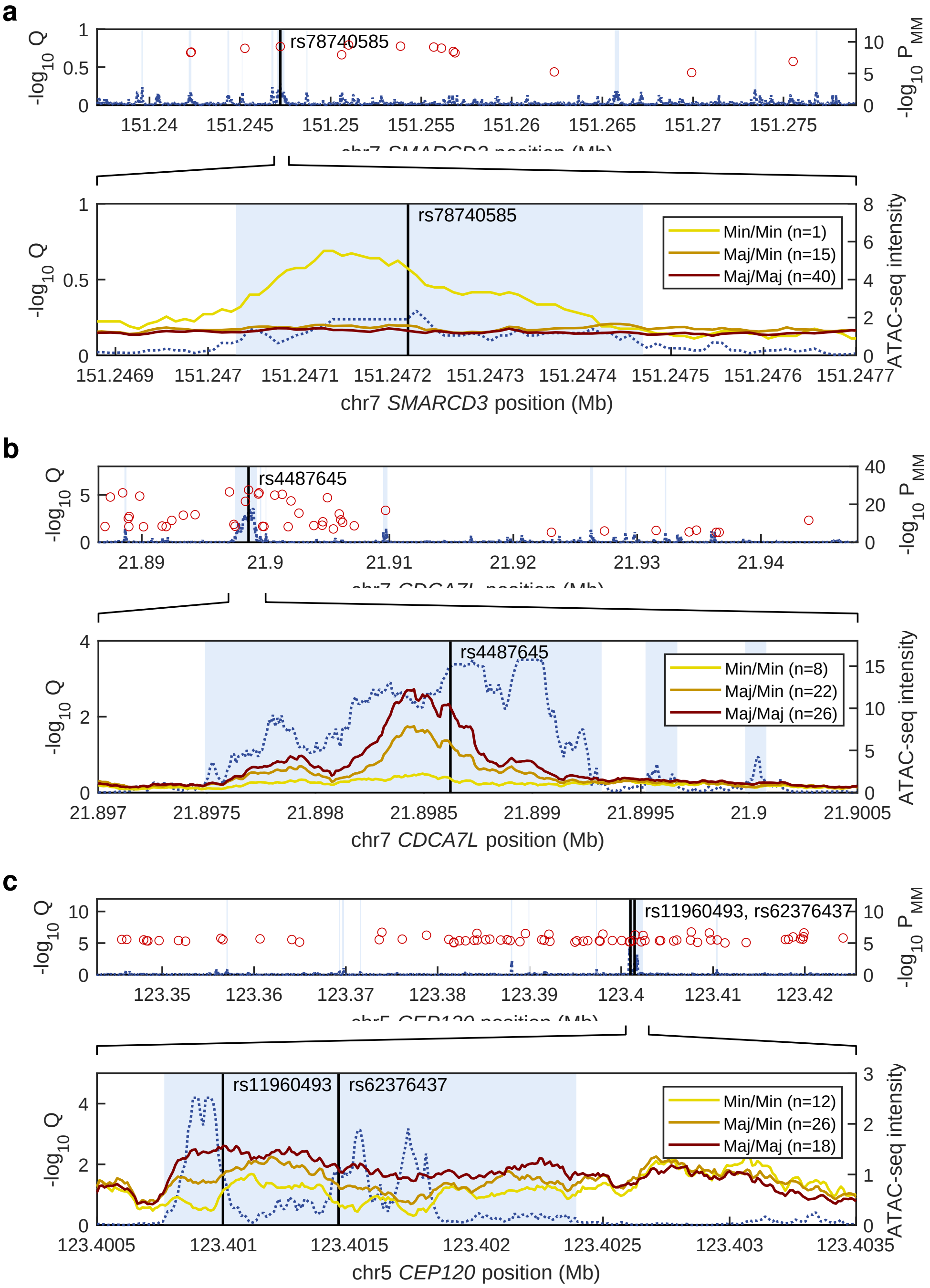

Supplementary Figure 12

Effect of varying λ_1 (1.025, 1.05, 1.075, 1.10, 1.125, 1.150, 1.175, and 1.20) while keeping λ_2 constant. The results shown are for the 56+105 MM plasma cell ATAC-seq samples. The parameter λ_1 serves to calibrate the cost of an allele-dependent model against the cost of an allele-independent model. Since an allele-dependent model is more flexible than an allele-independent model, it will always produce a lower L^2 residual, and λ_1 must therefore be greater than 1. Increasing λ_1 makes it more difficult to call a segment allele-dependent, yielding more conservative solutions.

Segmentation with λ_1=1.025, λ_2=0.001

SMARCD3

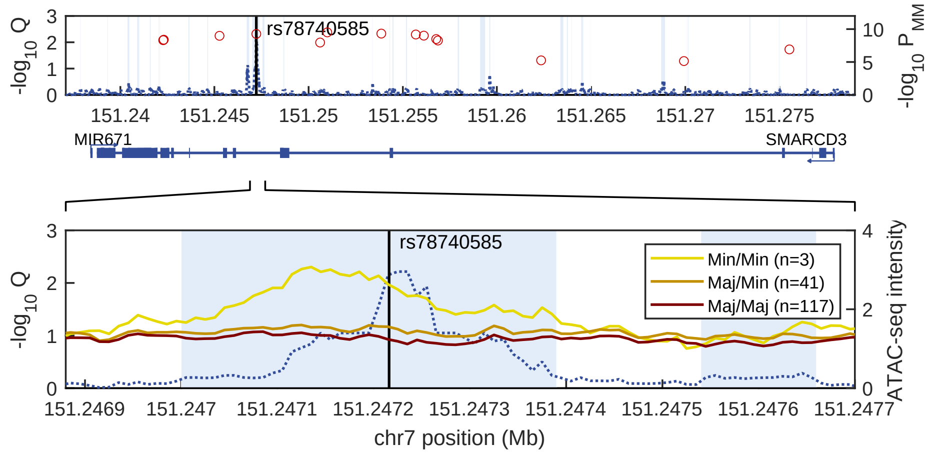

CDCA7L

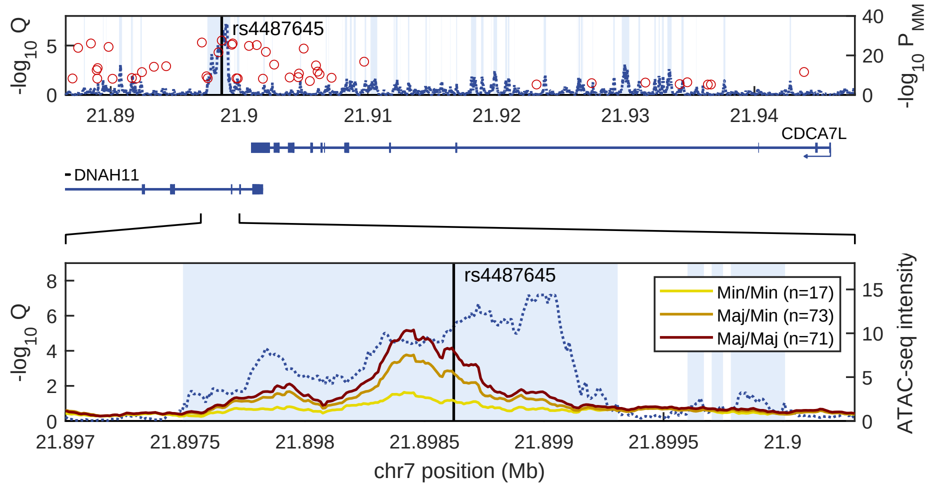

CEP120

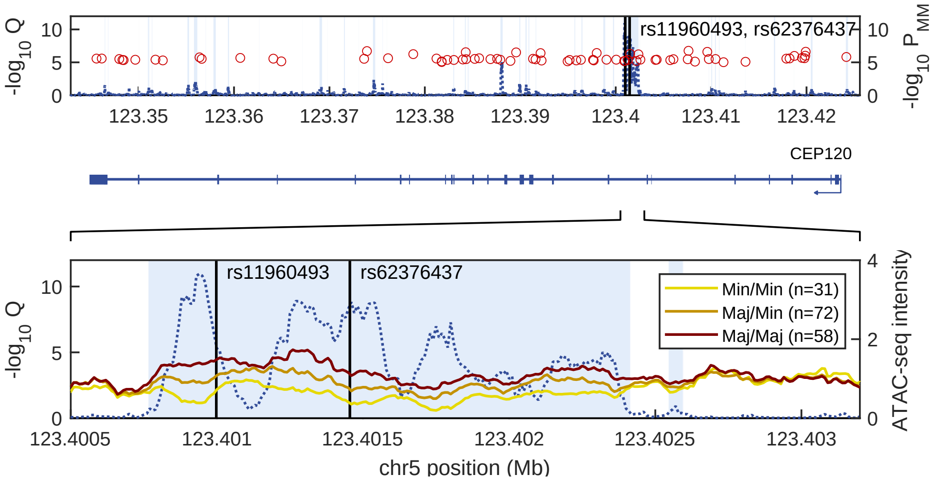

Segmentation with λ_1=1.05, λ_2=0.001

SMARCD3

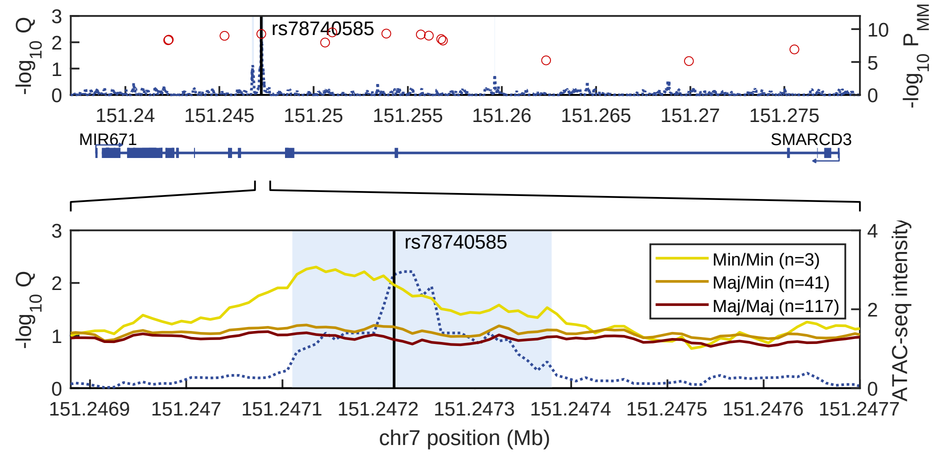

CDCA7L

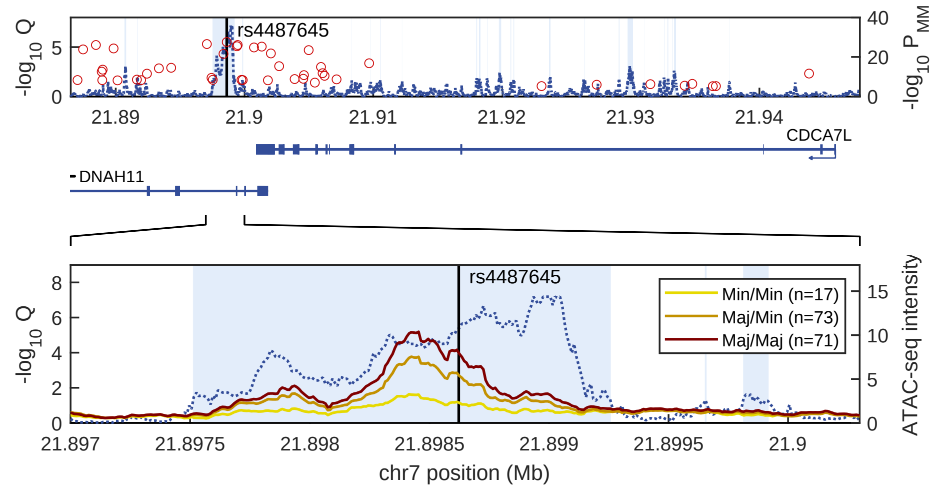

CEP120

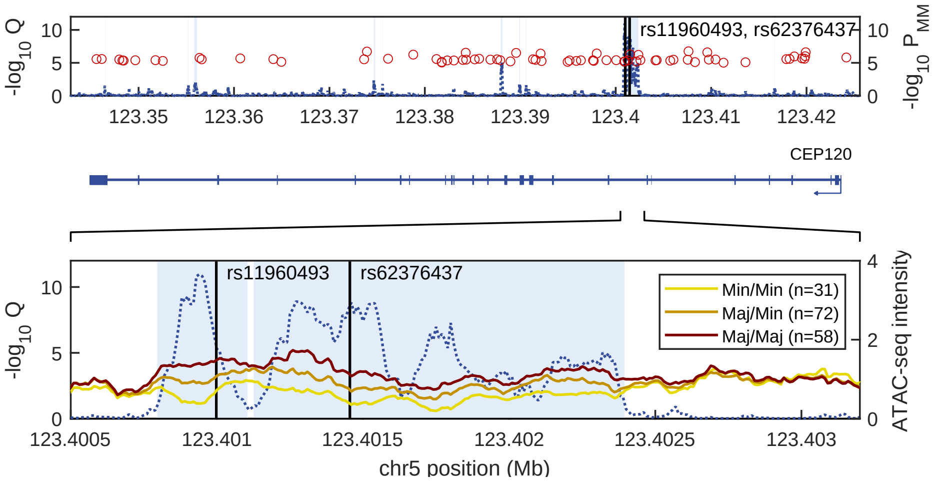

Segmentation with λ_1=1.075, λ_2=0.001

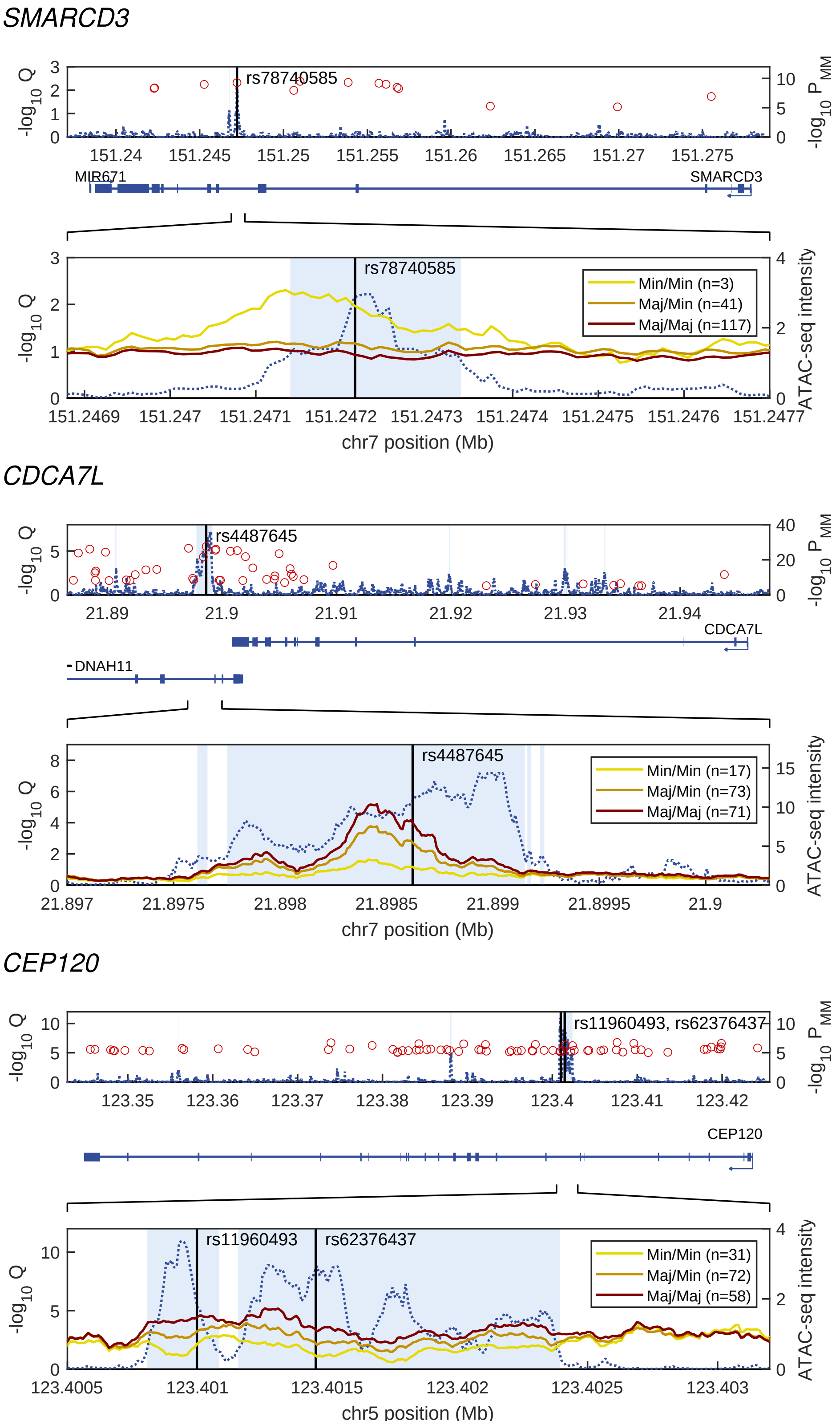

Segmentation with λ_1=1.1, λ_2=0.001

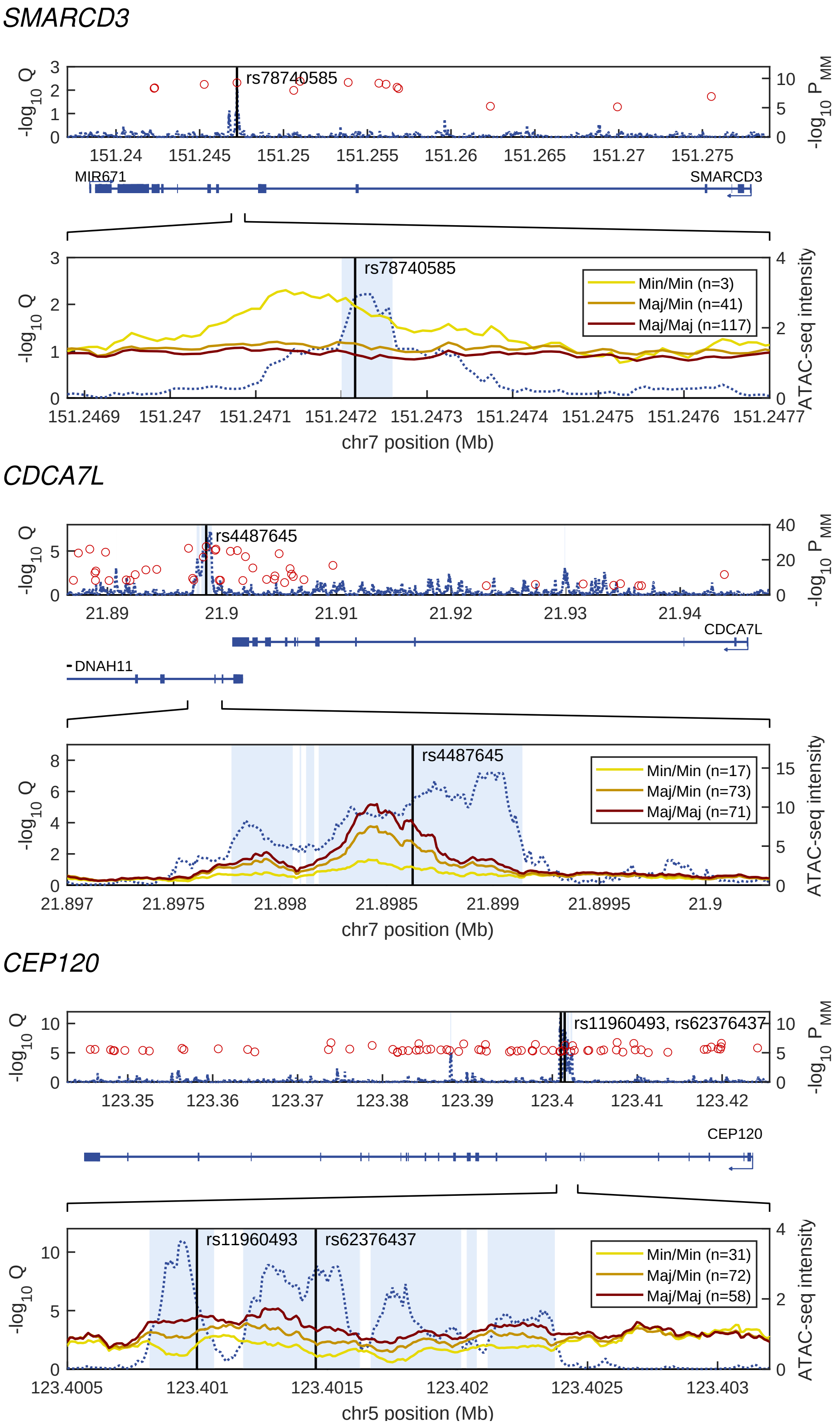

Segmentation with λ_1=1.125, λ_2=0.001

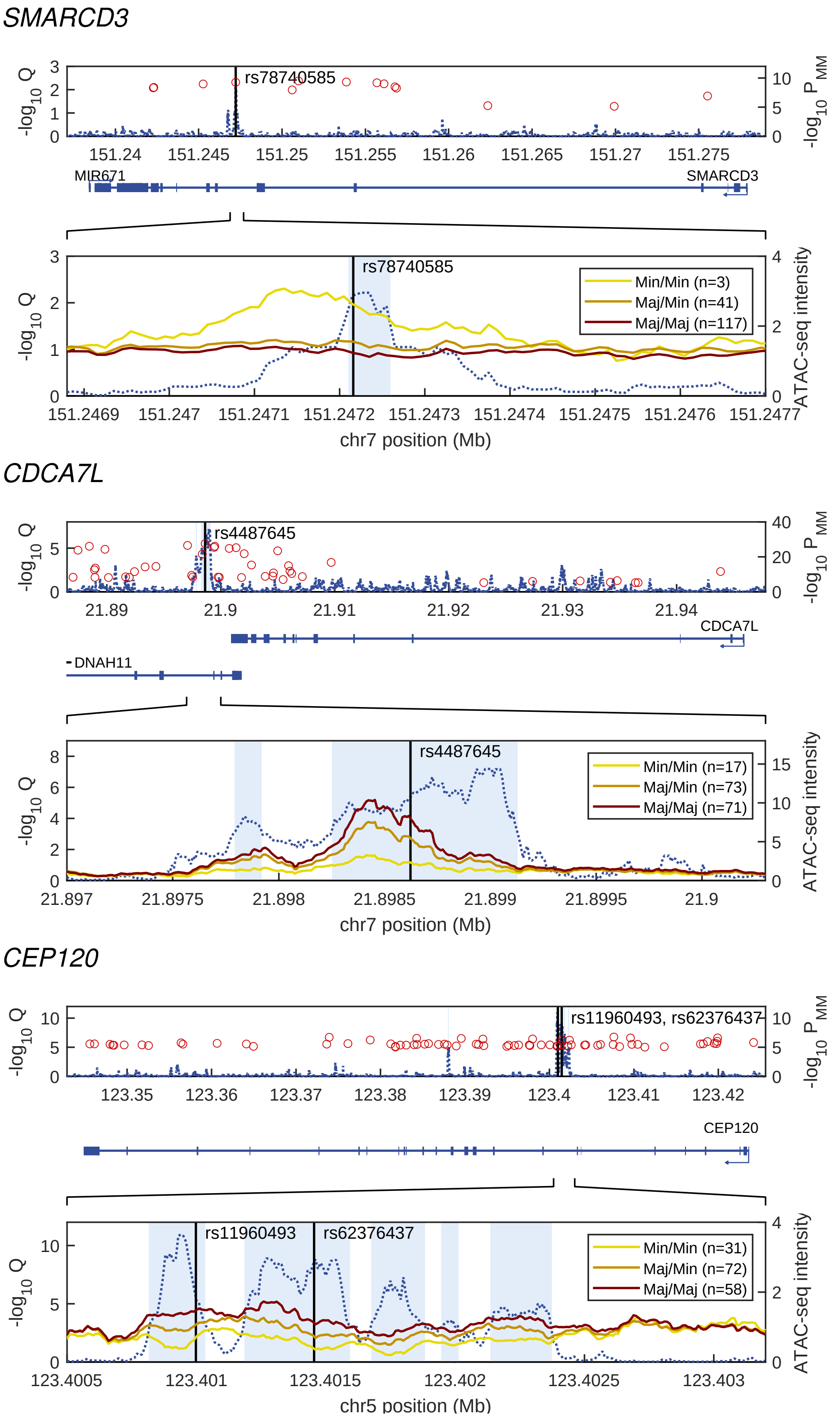

Segmentation with λ_1=1.15, λ_2=0.001

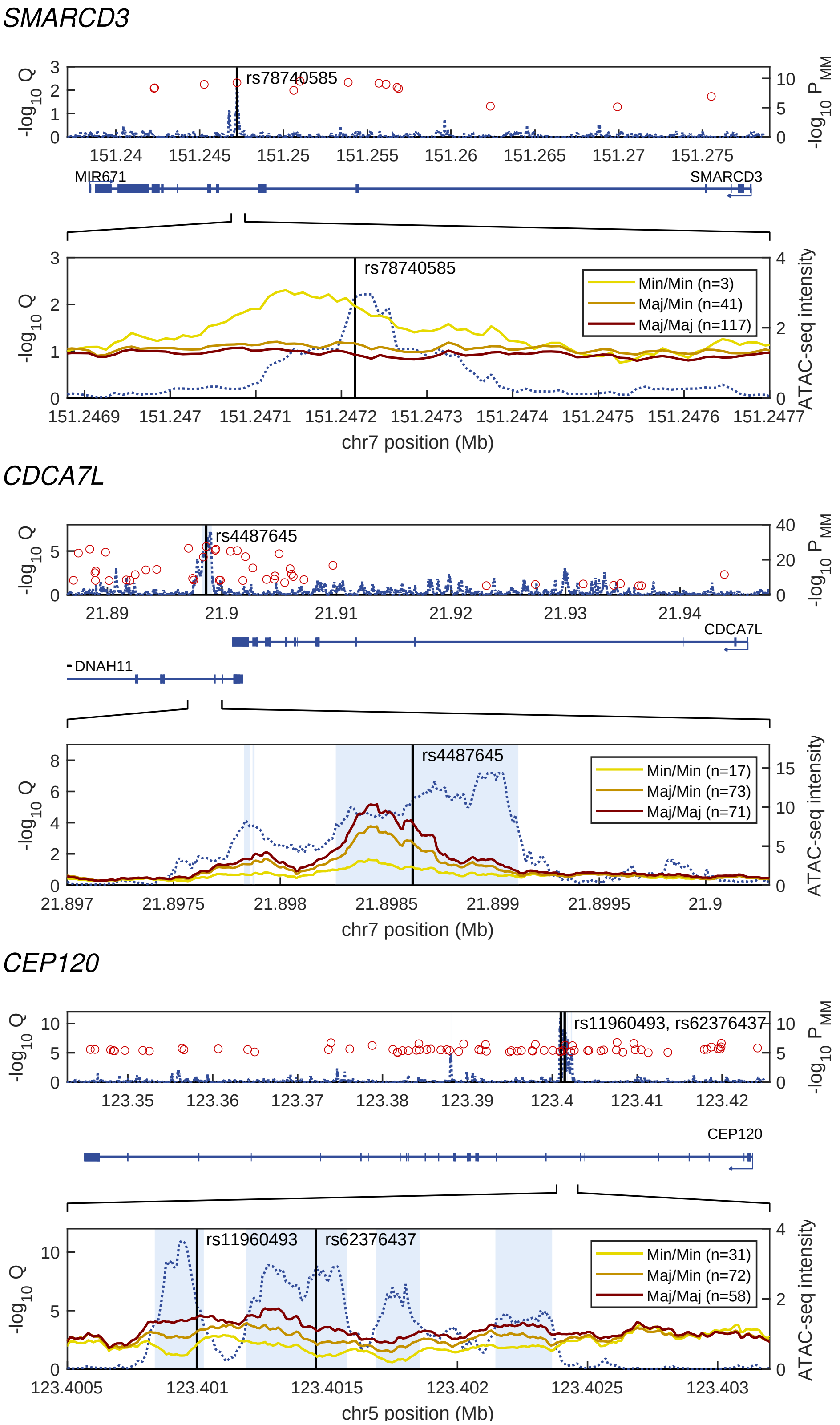

Segmentation with λ_1=1.175, λ_2=0.001

SMARCD3

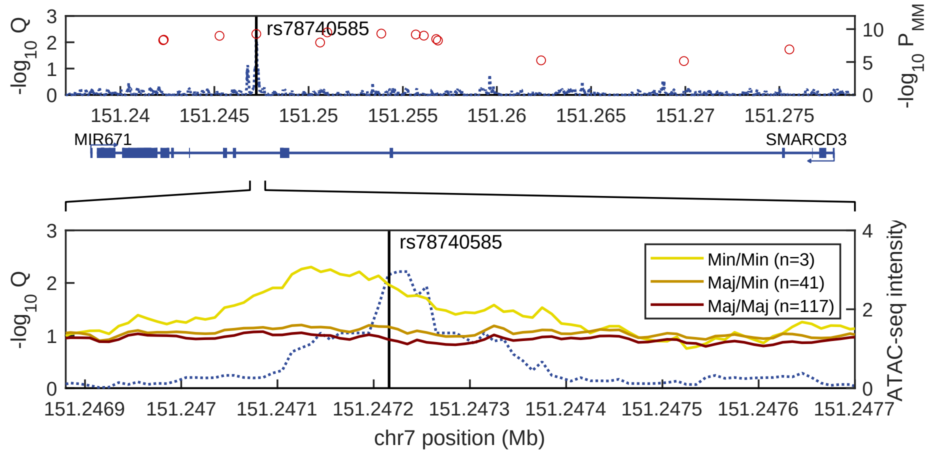

CDCA7L

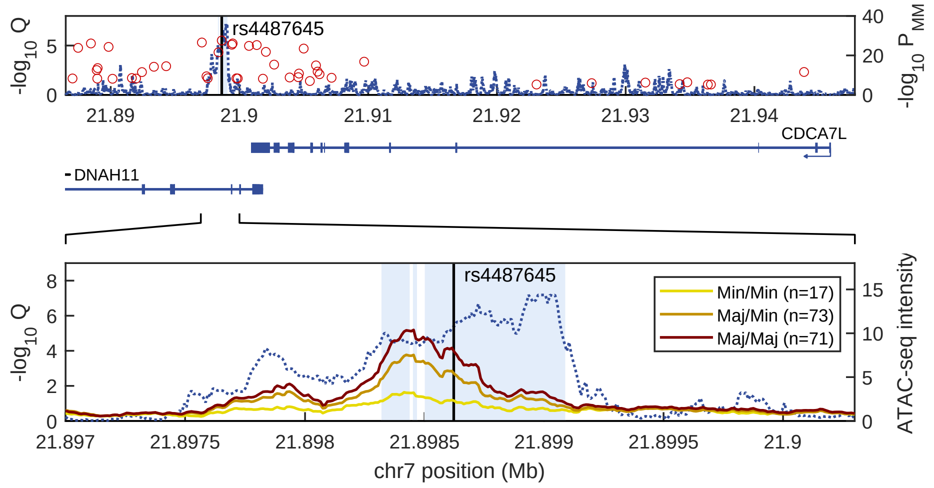

CEP120

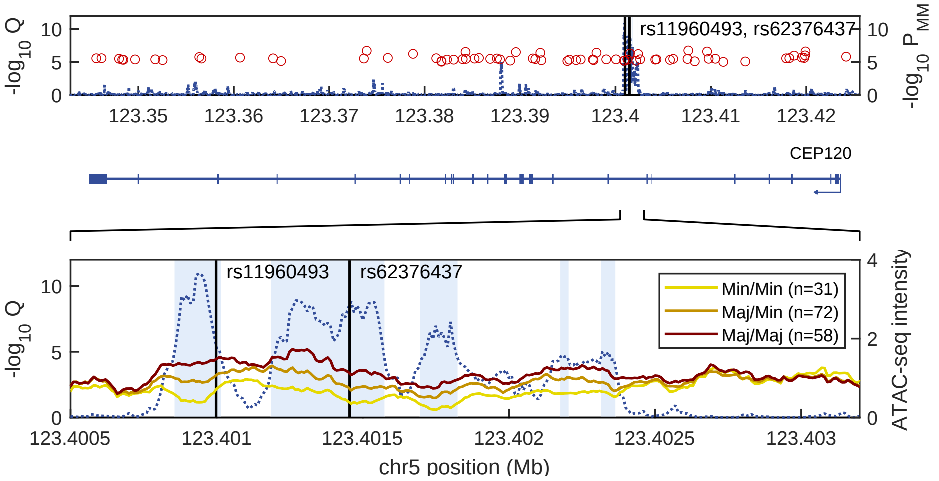

Segmentation with λ_1=1.2, λ_2=0.001

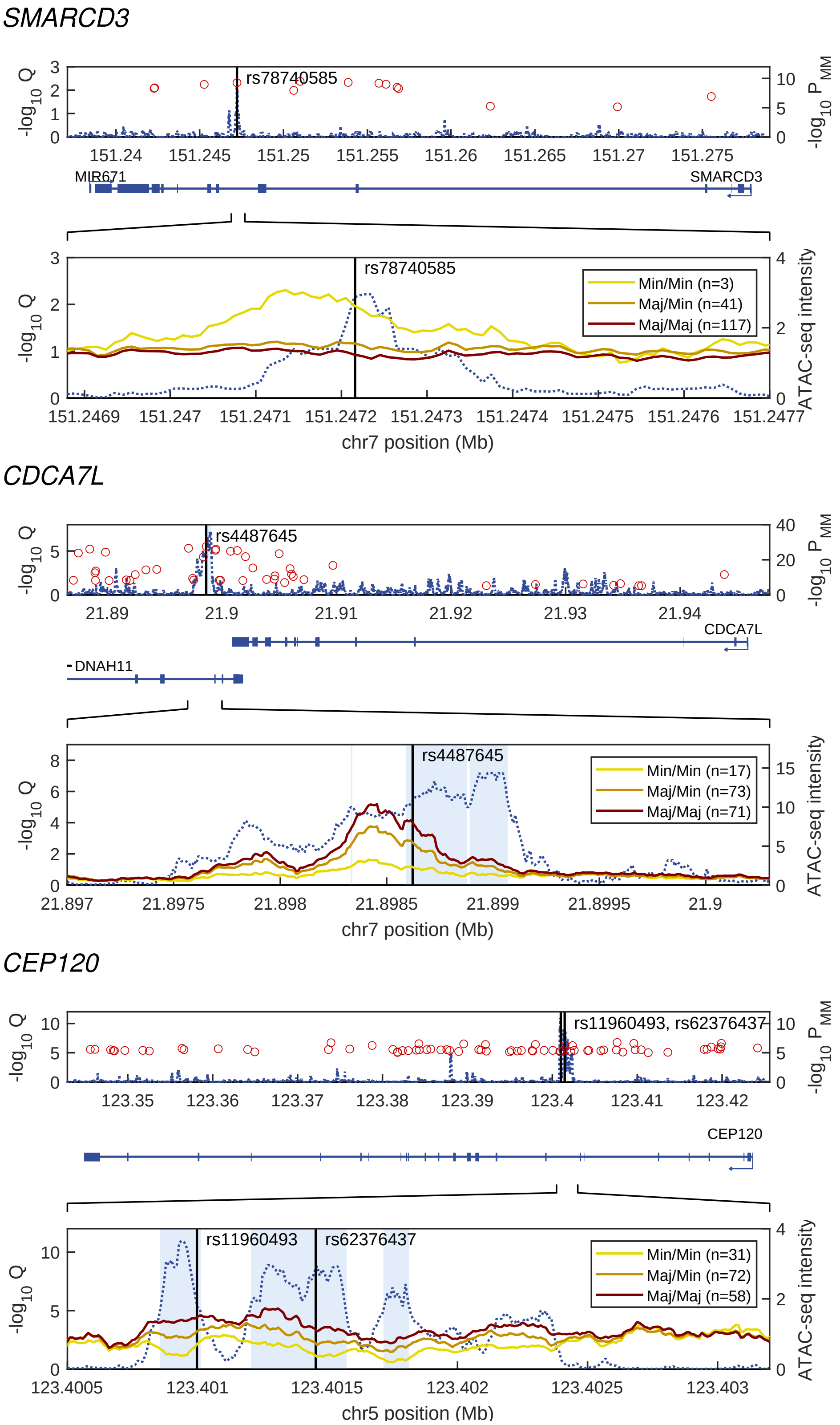

Supplementary Figure 13

Effect of varying λ_2 (0.00001, 0.0001, 0.001, 0.01, and 0.1) while keeping λ_1 constant. The results shown are for the 56+105 MM plasma cell ATAC-seq samples. The λ_2 parameter determines the cost of inserting an extra segment. Hence, increasing λ_2 produces a solution with fewer segments; decreasing λ_2 a solution with more segments.

Segmentation with λ_1=1.075, λ_2=0.00001

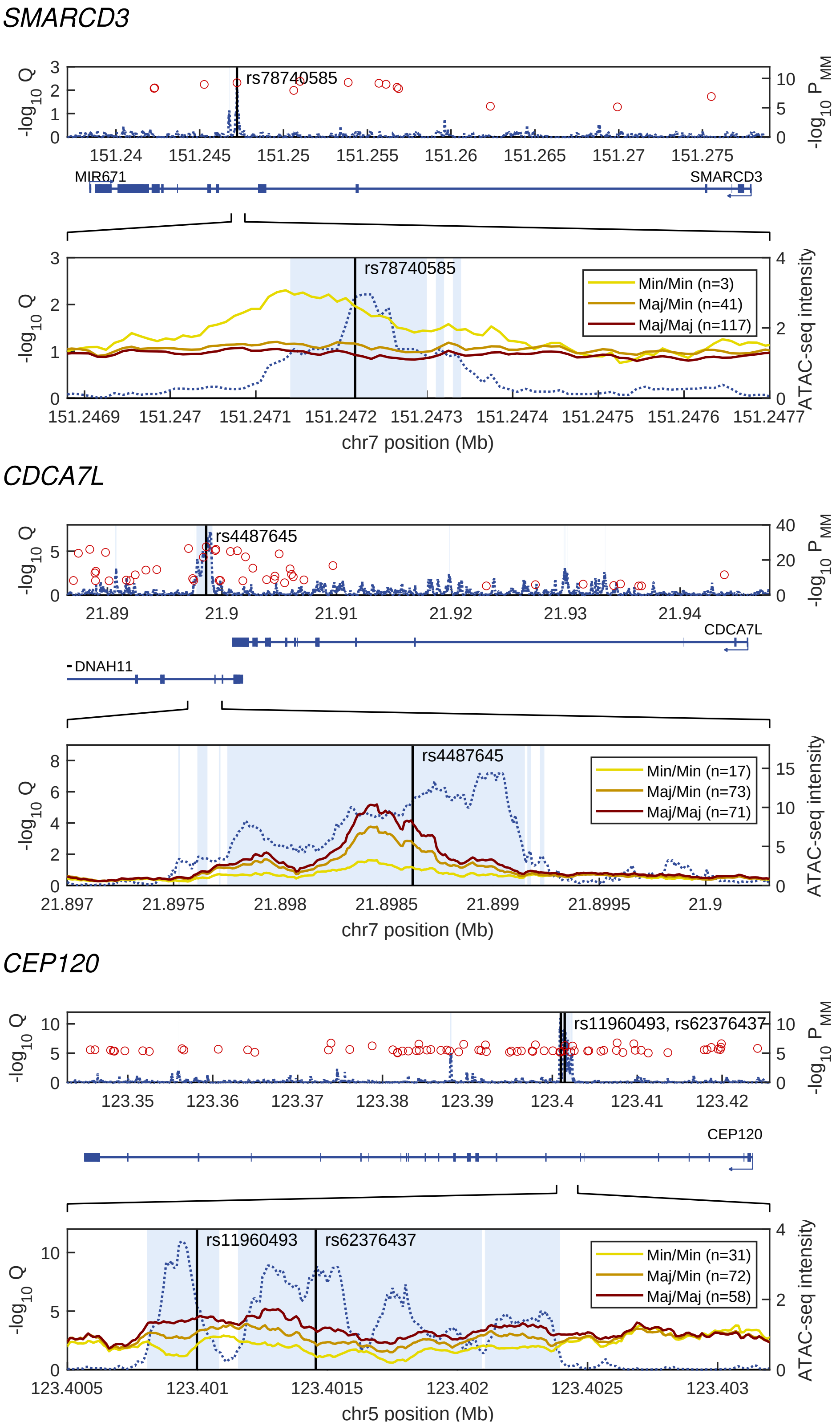

Segmentation with λ_1=1.075, λ_2=0.0001

SMARCD3

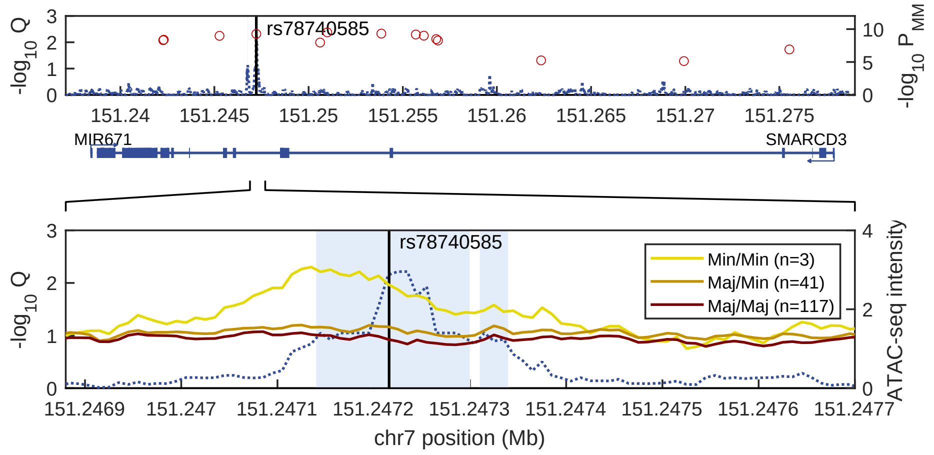

CDCA7L

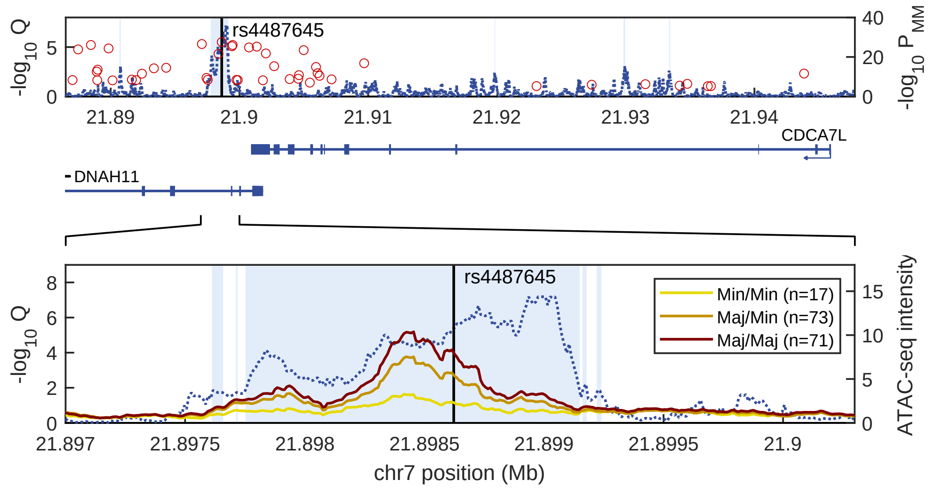

CEP120

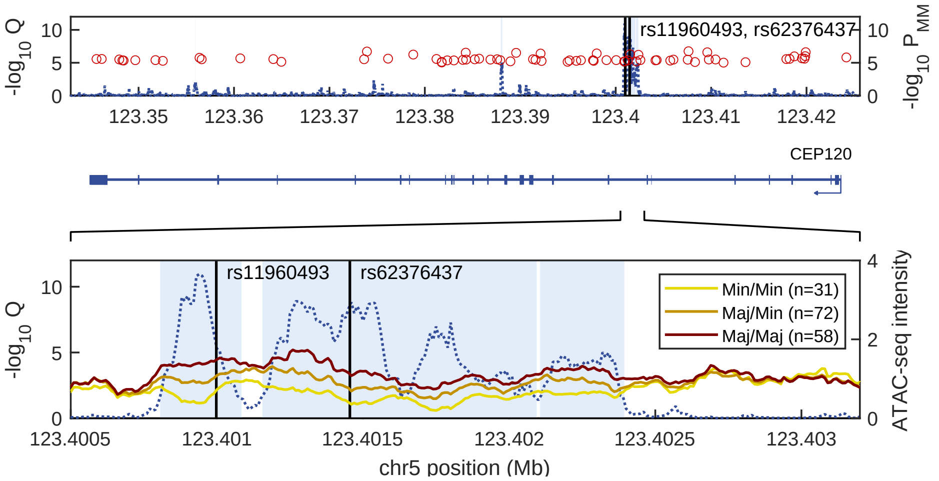

Segmentation with λ_1=1.075, λ_2=0.001

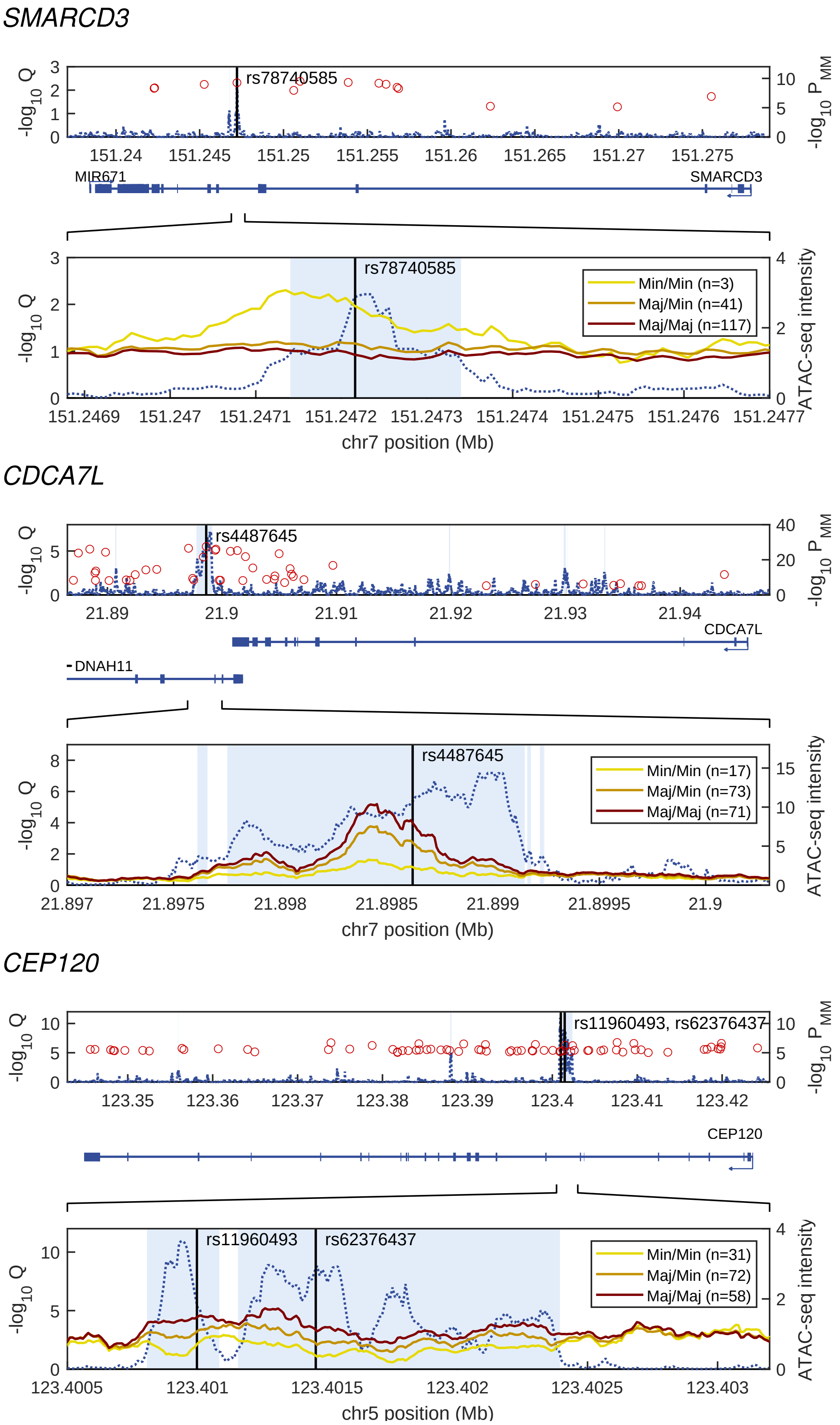

Segmentation with λ_1=1.075, λ_2=0.01

SMARCD3

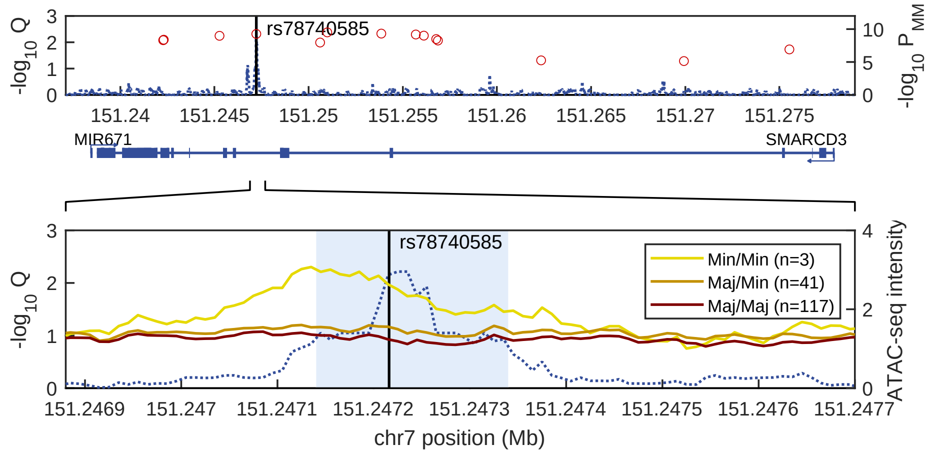

CDCA7L

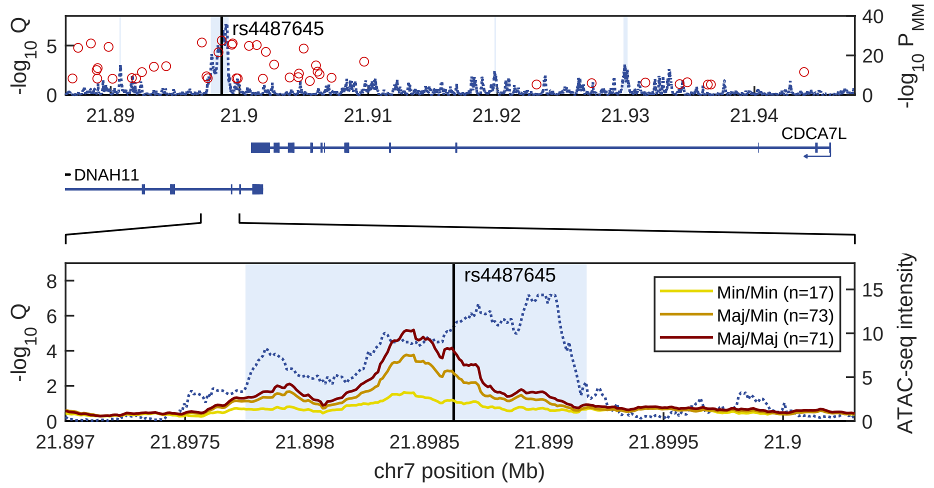

CEP120

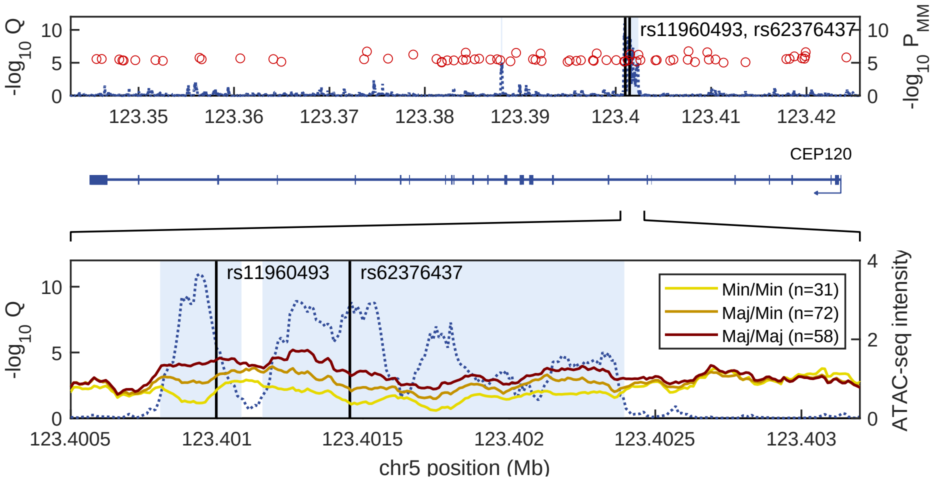

Segmentation with λ_1=1.075, λ_2=0.1

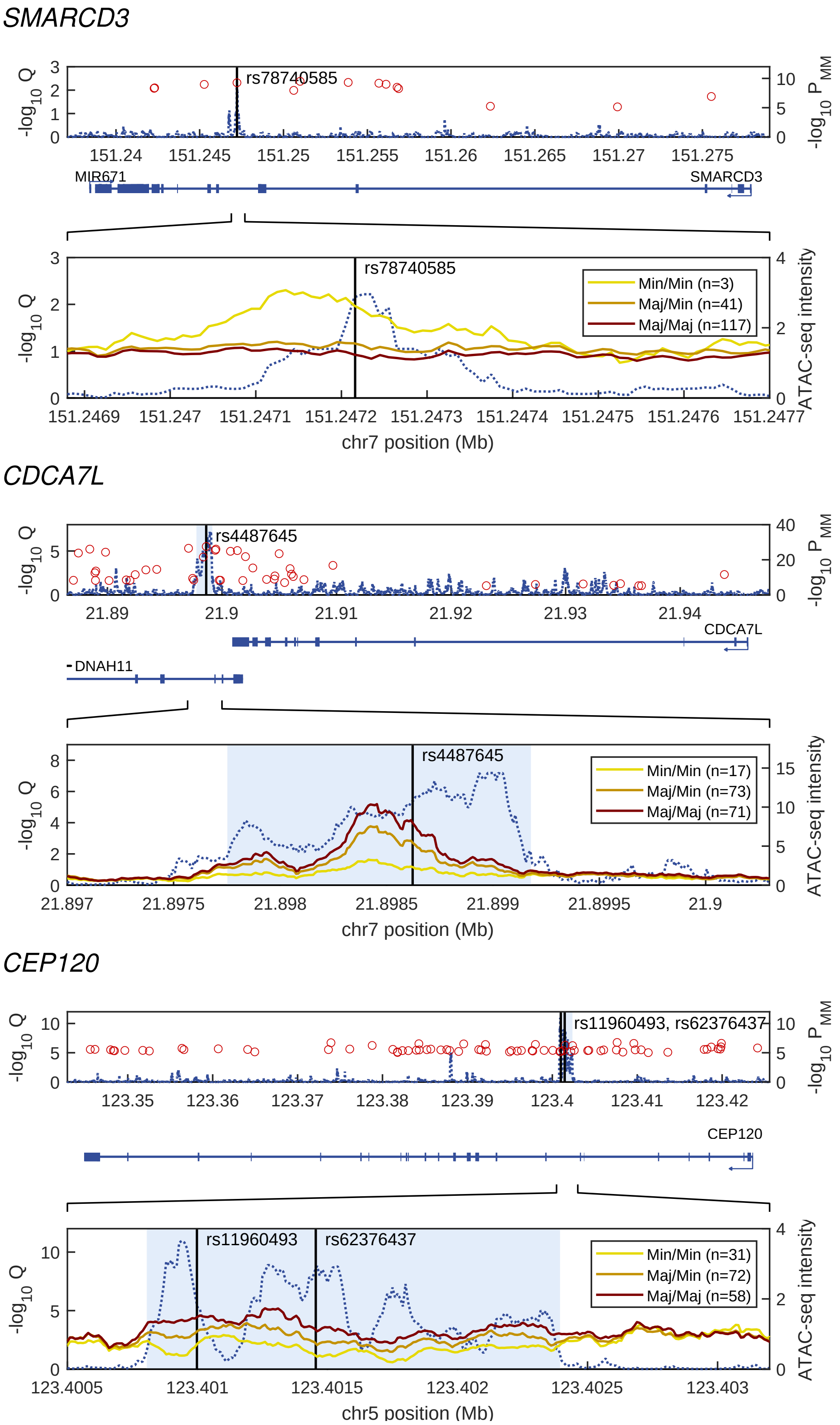

Supplementary Table 1

MM risk loci identified in well-controlled association studies. All associate with MM without further specification, except the *CCND1* variant, which selectively associates with MM with somatic t(11;14)[*IGH/CCND1*] translocation. In our MPRA, we included all loci except HLA and ULK4. Abbreviations: Risk Allele Frequency (RAF), Odds Ratio (OR). Underlined alleles are risk alleles. OR and *P*-values are for association with MM from the original publications.

						Discovery analysis			Latest meta-analysis[1]	
**Locus**	**Lead variant**	**Ref**	**Alt**	**RAF**	**Candidate genes**	**OR**	***P*-value**	**Ref**	**OR**	***P*-value**
2p23.3	rs6746082	A	C	0.76	*DNMT3A, DTNB*	1.29	$1.22x10^{-7}$	2		
	rs7577599	T	C	0.77		1.24	$1.24x10^{-16}$	3	1.23	$1.29x10^{-18}$
2q31.1	rs4325816	T	C	0.77	*SP3*	1.12	$7.37x10^{-9}$	1		
3p22.1	rs1052501	C	T	0.2	*ULK4*	1.32	$7.47x10^{-9}$	2		
	rs6599192	G	A	0.25		1.26	$8.75x10^{-18}$	3	1.26	$4.96x10^{-20}$
3q26.2	rs10936599	C	T	0.75	*TERC*	1.26	$8.70x10^{-14}$	4		
	rs10936600	A	T	0.75		1.2	$5.94x10^{-15}$	3	1.2	1.20x10-^{16}
5q15	rs56219066	T	C	0.71	*ELL2*	1.25	$9.6x10^{-10}$	5		
	rs1423269	A	G	0.71		1.17	$1.57x10^{-11}$	3	1.16	$8.30x10^{-12}$
5q23.2	rs6595443	A	T	0.48	*CEP120*	1.11	$1.20x10^{-8}$	1		
6p21.3	rs2285803	T	C	0.27	*HLA region*	1.19	$9.67x10^{-11}$	4		
	rs3132535	A	G	0.27	*HLA region*	1.2	$2.97x10^{-17}$	3	1.21	$6.00x10^{-21}$
6p22.3	rs34229995	C	G	0.03	*JARID2*	1.37	$1.31x10^{-8}$	3	1.36	$5.60x10^{-8}$
6q21	rs9372120	T	G	0.19	*ATG5*	1.18	$9.09x10^{-15}$	3	1.19	$2.40x10^{-15}$
7p15.3	rs4487645	C	A	0.65	*CDCA7L*	1.38	$3.33x10^{-15}$	2	1.24	$2.8x10^{-28}$
7q22.3	rs17507636	C	T	0.74	*CCDC71L*	1.12	$9.20x10^{-9}$	1		
7q31.33	rs58618031	T	C	0.75	*POT1*	1.12	$2.73x10^{-8}$	1		
7q36.1	rs7781265	G	A	0.09	*SMARCD3*	1.19	$9.71x10^{-9}$	3	1.22	$4.82x10^{-10}$
8q24.21	rs1948915	T	C	0.35	*CCAT1*	1.13	$4.20x10^{-11}$	3	1.15	$2.53x10^{-12}$
9p21.3	rs2811710	C	T	0.63	*CDKN2A*	1.15	$1.72x10^{-13}$	3	1.14	$3.64x10^{-11}$
10p12.1	rs2790457	G	A	0.73	*WAC*	1.12	$1.77x10^{-8}$	3	1.11	$2.66x10^{-6}$
11q13.3	rs603965 (rs9344)	G	A	0.51	*CCND1*	1.82	$7.95x10^{-11}$	6		
16p11.2	rs13338946	T	C	0.27	*several, including FBRS SRCAP, PRR14, RNF40*	1.15	$1.02x10^{-13}$	1		
16q23.1	rs7193541	T	C	0.61	*RFWD3*	1.13	$5.00x10^{-12}$	3	1.12	$3.68x10^{-10}$
17p11.2	rs4273077	A	G	0,11	*TNFRSF13B*	1.26	$7.67x10^{-9}$	4		
	rs34562254	G	A	0.1		1.3	$3.63 x 10^{-17}$	3	1.3	$1.18x10^{-19}$
19p13.11	rs11086029	T	A	0.25	*KLF2*	1.14	$6.79x10^{-11}$	1		
20q13.13	rs6066835	T	C	0.09	*PREX1*	1.26	$1.36x10^{-13}$	3	1.23	$6.58x10^{-10}$
22q13	rs138740	C	T	0.37	*TOM1*	1.18	$5.7x10^{-8}$	5		
	rs138747	A	T	0.36		1.21	$2.58x10^{-8}$	3	1.21	$2.58 x 10^{-8}$
22q13.1	rs877529	G	A	0.44	*CBX7*	1.23	$7.63x10^{-16}$	4		
	rs139402	T	C	0.44		1.23	$4.98x10^{-26}$	3	1.22	$3.84x10^{-26}$

References

1 Went, M. *et al.* I*Nature communications* **9**, 3707 (2018).
2 Broderick, P. *et al. Nature genetics* **44**, 58-61 (2012).
3 Mitchell, J. S. *et al. Nature communications* **7**, 12050 (2016).
4 Chubb, D. *et al. Nature genetics* **45**, 1221-1225 (2013).
5 Swaminathan, B. *et al. Nature communications* **6**, 7213 (2015).
6 Weinhold, N. *et al. Nature genetics* **45**, 522-525 (2013).

Supplementary Table 2

cis-eQTLs found within LD class of MM lead variants in MM plasma cells.

Locus	MM marker	Position (hg38)	eQTL marker	Position (hg38)	MM/eQTL marker correlation (r^2)	Other allele	Effect allele	eQTL gene	*P*-value	Effect
**Arkansas (n=1,445)**										**z-score**
7p15.3	rs4487645	21898622	rs4487645	21898622	1.00	C	A	*CDCA7L*	5.83E-39	-13.1
5q15	rs56219066	95907227	rs9314162	95916890	1.00	C	A	*ELL2*	1.82E-28	11.1
10p12.1	rs2790457	28567890	rs67343787	28552822	0.97	CAGTG	C	*WAC*	4.06E-26	10.6
7q22.3	rs17507636	106650672	rs73184243	106640054	0.87	G	A	*CCDC71L*	1.66E-10	-6.39
20q13.13	rs6066835	48738472	rs6066828	48727696	0.95	C	T	*PREX1*	5.70E-07	5.00
2q31.1	rs4325816	173944171	rs6715150	173890830	0.88	G	A	*SP3*	5.80E-04	-3.44
5q23.2	rs6595443	123407631	rs7706662	123419868	0.95	C	T	*CEP120*	6.94E-04	3.39
**CoMMpass (n=716)**										**β**
3p22.1	rs1052501	41883906	rs1052501	41883906	1.00	C	T	*ULK4*	1.03E-18	0.59
16q23.1	rs7193541	74630845	rs7193541	74630845	1.00	T	C	*RFWD3*	9.49E-16	-0.47
7q36.1	rs7781265	151253854	rs7782699	151218862	0.83	C	T	*SMARCD3*	9.23E-15	1.39
5q15	rs56219066	95907227	rs3815768	95900755	1.00	C	T	*ELL2*	2.64E-04	0.34
20q13.13	rs6066835	48738472	rs6122720	48734558	1.00	G	A	*PREX1*	1.70E-02	0.76
**Dana Farber (n=309)**										**β**
7q36.1	rs7781265	151253854	rs7782699	151218862	0.83	C	T	*SMARCD3*	3.16E-07	0.29
16q23.1	rs7193541	74630845	rs7193541	74630845	1.00	T	C	*RFWD3*	3.14E-04	-0.21
20q13.13	rs6066835	48738472	rs6122720	48734558	1.00	G	A	*PREX1*	6.85E-03	0.29
5q15	rs56219066	95920020	rs3815768	95900755	1.00	C	T	*ELL2*	9.97E-05	0.33
**Lund (n=185)**										**β**
10p12.1	rs2790457	28567890	rs2790457	28567890	1.00	G	A	*WAC*	8.61E-05	0.29
16q23.1	rs7193541	74630845	rs7193541	74630845	1.00	T	C	*RFWD3*	1.34E-03	-0.24
5q15	rs56219066	95907227	rs3815768	95900755	1.00	C	T	*ELL2*	7.00E-03	0.20
7q36.1	rs7781265	151253854	rs7782699	151218862	0.83	C	T	*SMARCD3*	7.78E-03	0.91

P-values are for regression analysis between genotype and expression of the eQTL gene.

Supplementary Table 3

cis-eQTLs found within LD class of MM lead variants in peripheral blood.

Locus	MM marker	Position (hg38)	eQTL marker	Position (hg38)	MM/eQTL marker correlation (r^2)	Other allele	Effect allele	eQTL gene	P value	Effect
**deCODE blood eQTL data (n=13,175)**										β
3p22.1	rs1052501	41883906	rs1052501	41883906	1.00	T	C	*ULK4*	0	1.32
3p22.1	rs1052501	41883906	rs1052501	41883906	1.00	T	C	*TRAK1*	1.05E-07	-0.10
3q26.2	rs10936600	169796797	rs10936600	169796797	1.00	A	T	*LRRC34*	2.36E-12	0.11
3q26.2	rs10936600	169796797	rs10936600	169796797	1.00	A	T	*ACTRT3*	8.68E-13	-0.11
5q23.2	rs6595443	123407631	rs6595443	123407631	1.00	A	T	*CEP120*	6.70E-26	0.13
5q15	rs56219066	95907227	rs1423269	95920020	0.96	A	G	*ELL2*	3.02E-08	-0.08
6p21.33	rs2285803	31139481	rs3132535	31148749	0.94	G	A	*CCHCR1*	0	-0.72
6p21.33	rs2285803	31139481	rs3132535	31148749	0.94	G	A	*POU5F1*	1.74E-24	0.14
6p21.33	rs2285803	31139481	rs3132535	31148749	0.94	G	A	*TCF19*	3.81E-63	-0.22
6q21	rs9372120	106219660	rs9372120	106219660	1.00	T	G	*ATG5*	5.37E-06	0.07
7p15.3	rs4487645	21898622	rs4487645	21898622	1.00	C	A	*CDCA7L*	7.96E-135	-0.33
7q36.1	rs7781265	151253854	rs7781265	151253854	1.00	G	A	*ABCF2*	4.04E-46	-0.25
9p21.3	rs2811710	21991924	rs2811710	21991924	1.00	C	T	*CDKN2A*	1.88E-05	0.05
9p21.3	rs2811710	21991924	rs2811710	21991924	1.00	C	T	*CDKN2B*	5.79E-91	-0.26
16p11.2	rs13338946	30689537	rs13338946	30689537	1.00	T	C	*FBRS*	2.16E-24	-0.14
16p11.2	rs13338946	30689537	rs13338946	30689537	1.00	T	C	*RNF40*	3.06E-64	0.24
16q23.1	rs7193541	74630845	rs7193541	74630845	1.00	T	C	*RFWD3*	7.57E-13	0.09
19p13.11	rs11086029	16327850	rs11086029	16327850	1.00	A	T	*KLF2*	5.44E-35	0.18
20q13.13	rs6066835	48738472	rs6066835	48738472	1.00	T	C	*PREX1*	9.77E-62	-0.34
22q13.1	rs139402	39150140	rs139402	39150140	1.00	T	C	*APOBEC3F*	1.10E-13	-0.09
22q13.1	rs139402	39150140	rs139402	39150140	1.00	T	C	*APOBEC3G*	3.62E-11	-0.08
22q13.1	rs139402	39150140	rs139402	39150140	1.00	T	C	*APOBEC3C*	3.89E-30	-0.14
22q13.1	rs139402	39150140	rs139402	39150140	1.00	T	C	*APOBEC3H*	4.36E-64	-0.22
22q13.1	rs139402	39150140	rs139402	39150140	1.00	T	C	*APOBEC3D*	7.41E-12	-0.09

eQTLGen blood eQTL data (n=31,684)										z-score
3p22.1	rs1052501	41883906	rs1052501	41883906	1.00	T	C	*ULK4*	3.27e-310	75.69
3p22.1	rs1052501	41883906	rs1052501	41883906	1.00	T	C	*TRAK1*	5.38E-12	-6.90
19p13.11	rs11086029	16327850	rs11086029	16327850	1.00	A	T	*KLF2*	1.13E-07	-5.30
16p11.2	rs13338946	30689537	rs13338946	30689537	1.00	T	C	*PRR14*	5.35E-36	-12.53
16p11.2	rs13338946	30689537	rs13338946	30689537	1.00	T	C	*RNF40*	2.61E-82	19.22
22q13.1	rs139402	39150140	rs139402	39150140	1.00	T	C	*CBX7*	9.63E-40	13.19
22q13.1	rs139402	39150140	rs139402	39150140	1.00	T	C	*APOBEC3F*	6.35E-07	-4.98
22q13.1	rs139402	39150140	rs139402	39150140	1.00	T	C	*APOBEC3H*	2.29E-103	-21.59
22q13.1	rs139402	39150140	rs139402	39150140	1.00	T	C	*APOBEC3G*	1.30E-28	-11.10
22q13.1	rs139402	39150140	rs139402	39150140	1.00	T	C	*APOBEC3C*	4.20E-51	-15.04
22q13.1	rs139402	39150140	rs139402	39150140	1.00	T	C	*APOBEC3D*	7.99E-26	-10.51
6p21.33	rs2285803	31139481	rs2285803	31139481	1.00	C	T	*TCF19*	3.59E-65	-17.05
6p21.33	rs2285803	31139481	rs2285803	31139481	1.00	C	T	*CCHCR1*	1.78E-44	-13.99
10p12.1	rs2790457	28567890	rs2790457	28567890	1.00	G	A	*WAC-AS1*	8.99E-279	-35.68
9p21.3	rs2811710	21991924	rs2811710	21991924	1.00	C	T	*CDKN2B*	1.48E-79	-18.89
9p21.3	rs2811710	21991924	rs2811710	21991924	1.00	C	T	*CDKN2A*	2.89E-15	7.90
7p15.3	rs4487645	21898622	rs4487645	21898622	1.00	C	A	*CDCA7L*	2.96E-209	-30.87
5q15	rs56219066	95907227	rs56219066	95907227	1.00	T	C	*ELL2*	1.47E-36	-12.63
20q13.13	rs6066835	48738472	rs6066835	48738472	1.00	T	C	*PREX1*	9.23E-84	-19.39
16q23.1	rs7193541	74630845	rs7193541	74630845	1.00	T	C	*RFWD3*	6.93E-102	21.43

P -values are for regression analysis between genotype and expression of the eQTL gene.

Supplementary Table 4

cis -eQTLs found within LD class of MM lead variants in B cells.

Locus	MM marker	Position (hg38)	eQTL marker	Position (hg38)	MM/eQTL marker correlation (r^2)	Other allele	Effect allele	eQTL gene	P value	Effect
**deCODE B cell eQTL data (n=758)**										β
3p22.1	rs1052501	41883906	rs1052501	41883906	1.00	T	C	*ULK4*	4.15E-41	0.97
6p21.33	rs2285803	31139481	rs2285803	31139481	1.00	C	T	*CCHCR1*	5.05E-72	-0.9068
6p21.33	rs2285803	31139481	rs2285803	31139481	1.00	C	T	*HLA-C*	4.24E-17	-0.45991
6p21.33	rs2285803	31139481	rs3132535	31148749	1.00	G	A	*C4B*	7.09E-14	0.41104
6p21.33	rs2285803	31139481	rs2285803	31139481	1.00	C	T	*APOL4*	1.49E-13	0.40633
6p21.33	rs2285803	31139481	rs2285803	31139481	1.00	C	T	*HCG27*	4.59E-13	-0.39833
6p21.33	rs2285803	31139481	rs3132535	31148749	1.00	G	A	*HLA-DRB4*	2.75E-11	-0.36704
6p21.33	rs2285803	31139481	rs3132535	31148749	1.00	G	A	*HLA-DRB6*	6.50E-10	-0.34119
6p21.33	rs2285803	31139481	rs3132535	31148749	1.00	G	A	*TCF19*	1.43E-09	-0.3344
6p21.33	rs2285803	31139481	rs2285803	31139481	1.00	C	T	*HLA-DRB5*	4.66E-09	0.32439
6p21.33	rs2285803	31139481	rs2285803	31139481	1.00	C	T	*C4A*	1.25E-08	0.31537
6p21.33	rs2285803	31139481	rs3132535	31148749	1.00	G	A	*ATP6V1G2*	2.22E-08	-0.30962
6p21.33	rs2285803	31139481	rs3132535	31148749	1.00	G	A	*HLA-DRB1*	5.55E-08	-0.30089
6p21.33	rs2285803	31139481	rs3132535	31148749	1.00	G	A	*ZNF843*	5.12E-06	-0.25327
6p21.33	rs2285803	31139481	rs3132535	31148749	1.00	G	A	*ASRGL1*	7.78E-06	0.24842
6p21.33	rs2285803	31139481	rs2285803	31139481	1.00	C	T	*TPD52*	9.38E-06	0.24652
16p11.2	rs13338946	30689537	rs13338946	30689537	1.00	T	C	*CCDC189*	7.21E-15	-0.45683
16q23.1	rs7193541	74630845	rs7193541	74630845	1.00	T	C	*RFWD3*	1.21E-06	-0.25575
17p11.2	rs4273077	16945825	rs4273077	16945825	1.00	A	G	*TNFRSF13B*	8.93E-06	0.43996
19p13.11	rs11086029	16327850	rs11086029	16327849	1.00	A	T	*KLF2*	5.90E-13	0.43109
22q13.1	rs139402	39150140	rs877529	39146287	1.00	G	A	*APOBEC3D*	5.09E-07	-0.25988

P -values are for regression analysis between genotype and expression of the eQTL gene.

Supplementary Table 5

Sequences used in luciferase experiments. Reference/alternative nucleotides within brackets. We performed luciferase experiments 20 variants, including 18 that were significant in both cell lines and the top-ranking MPRA variants at the *RFWD3* and *CCDC71L* loci.

rs3777189

TGAGCATCTGTTTTTACCCTTTTATCTCCAGGTTGAGTCAATAAGCATGTATTTTGAGCT**[C/G]**AGTCAGCACTGTGAAATTAGAAAAAGGGCATAAGAAAGCCTCTGACCTTGAAGAGCATGC

rs78740585

GCCCAGGCCTGGCCACCAGCTAGGTGGGGCACTGGGGCTCACAGCCACTCAAGAACTGAA**[G/A]**CTGTAAGTTGACCAGATTAAAAAAACAGTCCACGTGTACAGCCTCTCCCCACTAGATGG

rs188468174

GGACCAGGCCTTCCCAGTGTCAACCCAACCTCAGCTCACAGGATGCGAGAAGCCTGCTCG**[C/T]**GGCCTTGGCTCATTGGCTGGGCCGCGGTCACCTGGGCCGTGATGTCACGGCCTTTTAGAA

rs4273077

AATGGCCTTGAATTCACCATGGCTACAGGTTTCCCCCCTCCCCCAGCTTGCTCCTTCC**[A/G]**CCAAGAGGTGGCGTCTGTTTCCCCTCCCCTGAATCCGCTGGCACTGTGAATGGAAAGAA

rs11711621

CAGTTCAATGGGAGAAAGAATAGTCTTTTAACAAATGGTGTTAGGACACGTGGATATACA**[C/T]**ATGCAAAAGAATAAAGTTGGATTCCTACCTCATACCATATTCAAAAATTAAAATGGATCA

rs3827644

TATTTCTTTAATCCTTTAAAATTCAAATCACACAATGCTCCAATGAAATCTTCATTAACT**[G/C]**AACCAAACTATGCCCATGAAAGATCTCATATGCAACTGCTAAAACCTCAATAAACATAT

rs6066831

AACGGAGAGGCTAAAGGGCCCTCAGACCAGCTCTGTGCTTCTTCCCAGGCTGGTCCCACC**[C/A]**CACCCCAGGGACCATAAGTGAGCACATGACCCAGACAAAACCAATCAGAGTTCCCCGTCC

rs4792800

TGTTCATCTCTTTTCTGTCTCTGTGGATTTGCCTATTCCAGACATTTCATATTAATGAA**[A/G]**TCATACAACACGTGGTCTTTTATGCCTGGCTCCTTGCACTTGGCTTTGAGTTTTCAAGG

rs13198600

CTCTTGCAAATCTCAGTTGGCTCGAAATAGCTGTTCTATGTTCTTTCAATGGAAGCCCCC**[A/G]**CCTTCTGAGTACACTGGTTCATAGTTATTTATAAACCTGATGTCACTCAAATATTATTT

rs2790444

AACAAAACCAATTCTCAGTAACTATTTTCAGACTTAATTTGCC**[C/T]**GAATTACATTTCATGTCAAAAGCATGCATTAAGGAACAAAGCAATTTCAGTAAAGACACCTATATTCTCTCCTACA

rs77791277
CCTGGAAGACTTGGAGCCACTGTCTGTCGGACTTACTTCACCCATACTGGTATGTCTGA**[G/A]**GGGGGTGGAGTTCAAGATCAGGCAAGAGAAAAACATGTAAAGGAAGTAACCTCCCAAC

rs4487645
TTTAAGCAGCCTCTGAAACTTACAATTCAAGGTTTCACTT**[C/A]**TCTCTTAATTTTATCGAAGAGGTTCATCTGTCCCTAGCCTCTGTGAGCCAGCTTCCTTAT

rs11960493
GAGGACTCGGACACCAGCTTCCCATCACGTGTCTCGATCTTCACAACCACGGCCCTGGAG**[T/G]**AGCTGGTGCGGCTGAAGGAGCTGGCGCCCAGGCCGTAGCTGAGGCCGGGGCTTGTGAGGC

rs11241694
GGCACAGGCTCAGTAAAAGACTGAGACCTAACCATAAGGCTATAGAATGCTTCCCCTTCC**[T/C]**GACTCCTGACCACTACATTACTAAGGCCTATTTACAGCAGTTCCTTTTGTCCCATACATC

rs3777183
TTCAAAGGGAGTTTTCTTATGATTCATTTTTGTAGCTTCATGGCTAAAAAGTATTTAGAA**[G/A]**AACACAGGTGTCTGAGTGTGTGTTGTTTGGTAGTTGGGTATTTCCAGGAAAAAAGGGGTG

rs3777182
TATGATTCATTTTTGTAGCTTCATGGCTAAAAAGTATTTAGAAGAACACAGGTGTCTGAG**[T/A]**GTGTGTTGTTTGGTAGTTGGGTATTTCCAGGAAAAAAGGGGTGATGACAGTTACTGGCAG

rs3804329
TCCTATTAACACACCAGCTCTCCTGAGCTAAGCAGCTCCTGGGGTTGGGGAAGGGTGTAC**[A/G]**TGGAGAAAGCTAGAACCTCTACAGTGTTTTCCTCTCTGGGAGGAACTAGCAGGCATACG

rs6066832
ACGGAGAGGCTAAAGGGCCCTCAGACCAGCTCTGTGCTTCTTCCCAGGCTGGTCCCACCC**[C/A]**ACCCCAGGGACCATAAGTGAGCACATGACCCAGACAAAACCAATCAGAGTTCCCCGTCC

rs8051148
TCTCGGAACACTGTTTTTGTTTTTTTTGTTTTTTTTTTTTGAGATGAAGTCTCGCTCTTG**[T/C]**TCCCCAGGCTGGAGTGCGATGGCGCGATCTCGGCTCACTGCAACCTCTGCCACCCGGGT

rs73184243
AATCCCAGCAAAACCAAGATGGTGACAAAAGTTACCTCTGGTCTTCACTGCTCATTATAC**[G/A]**ATAATTATATAATGCATTAGCATGCTAAAAGACACTCCCACCAGTGCCATTGACAGTTT

Supplementary Table 6

Plasma cell meQTLs detected for the selected MPRA-functional variants (n=379).

Genomic position				Methylation		
**Chrom**	**Pos**	**rsID**	**Gene**	**Probe**	**P-value**	**Effect**
chr20	47354132	rs6066832	*PREX1*	cg18249380	4.64E-05	0.052
chr10	28825689	rs2790444	*WAC*	cg11724873	1.37E-08	-0.051

P -values are for linear correlation between genotype and methylation.

Supplementary Table 7

Probes and primers used for MPRA.

MPRA_v3_Illumina_GFP_F
GTGACTGGAGTTCAGACGTGTGCTCTTCCGATCTCGCCCTGAGCAAAGACC

Illumina_Universal_Adapter
AATGATACGGCGACCACCGAGATCTACACTCTTTCCCTACACGACGCTCTTCCGATCT

MPRA_v3_Amp2Sc_R
CCGACTAGCTTGGCCGC

GFP_BiotinCapture_1
CGCCGTAGGTGAAGGTGGTCACGAGGGTGGGCCAG/3BioTEG/

GFP_BiotinCapture_2
CCAGGATGTTGCCGTCCTCCTTGAAGTCGATGCCC/3BioTEG/

GFP_BiotinCapture_3
CCTCGATGTTGTGGCGGGTCTTGAAGTTCACCTTG/3BioTEG/

MPRA_v3_F
GCCAGAACATTTCTCTGGCCTAACTGGCCGCTTGACG

MPRA_v3_20I_R
CCGACTAGCTTGGCCGCCGACGCTCTTCCGATCT(N1:25252525)(N1)(N1)(N1)(N1)(N1)(N1)(N1)(N1)(N1)(N1)(N1)…
...(N1)(N1)(N1)(N1)(N1)(N1)(N1)(N1)TCTAGAGGTTCGTCGACGCGATCGCAGGAGCCGCAGTG

MPRA_v3_GFP_Fusion_v2_F
CACTGCGGCTCCTGCGATCTAACTGGCCGGTACCTGAGCTCGCTA

MPRA_v3_GFP_Fusion_v2_R
TCTAGAGGTTCGTCGACGCGATTATTATCATTACTTGTACAGCTCGTCCATGC

MPRA_v3_Amp2Sa_Illumina_F
GTGACTGGAGTTCAGACGTGTGCTCTTCCGATCTAACTGGCCGCTTGACG

Illumina_Multiplex
CAAGCAGAAGACGGCATACGAGATCAGAATAGTCGTGACTGGAGTTCAGACGTGTGC (10 bp barcode, R1(E2C97))

Supplementary Table 8

Guide sequences used in dual-sgRNA CRISPR/Cas9 deletion experiments.

**rsID**	**gene**	**sgRNA sequence**
rs3777189	*ELL2*	ACATGCTTATTGACTCAACC
rs3777189	*ELL2*	ACCTTGAAGAGCATGCACTG
rs3777183-rs3777182	*ELL2*	TGGTTTCGTCAGCTAAAAAG
rs3777183-rs3777182	*ELL2*	GGTGATGACAGTTACTGGCA
rS4487645	*CDCA7L*	GTTTCAGAGGCTGCTTAAAG
rS4487645	*CDCA7L*	AAGCTGGCTCACAGAGGCTA
rs2790444	*WAC*	CTCCTACCACAAAGAGCTTG
rs2790444	*WAC*	CAACATGTAGGAGAGAATAT

Supplementary Table 9

Amplification primers to confirm CRISPR/Cas9 deletions.

Gene	Primer sequence	Targeted region
WAC_F	GGAACTTTGACCTTTTAAAGATTTCAGTTTGAGATC	rs2790444
WAC_R	GAACTACTTTCTTCTCATCCAAATTATTCAATCCCAG	
CDCA7L_F	TCCTTTGTGTGGTGCCTATGCTG	rS4487645
CDCA7L_R	ATCACCCTATGACTTGGACTGACAAGC	
ELL2_F	AGGCAAGAAGGTACTCTCTAGAATTC	rs3777189
ELL2_R	TGGTTATTTCTACCTTCCTTGTTTG	
ELL2_F	TGCAGGTACAGGCAGCAGGATTCC	rs3777183-rs3777182
ELL2_R	TCTCGATGGCTGTCTCACTTCTCTTCC	

Supplementary Table 10

qPCR primers used for readout in the CRISPR/Cas9 deletion experiments.

Primer	Sequence
WAC_F	AGCAAGGACCAGTGTCACAGTC
WAC_R	GACCAGGTGATGGACTTCTCTG
CDCA7L_F	TTGGCGGAATTGAACTCGATGCC
CDCA7L_R	GTTCATACGCCGCGTGATCTGT
ELL2_F	CACCAGCCGTTCAGAATCTCCT
ELL2_R	GGTGGTACTCTGTTCGTCAGGT
GAPDH_F	GTCTCCTCTGACTTCAACAGCG
GAPDH_R	ACCACCCTGTTGCTGTAGCCAA

Supplementary Table 11

Percent of region-of-interest called allele-dependent with different λ_1 and λ_2. Estimated noise proportion (π_0) within brackets.

***SMARCD3* rs78740585**

	$\log_{10}(\lambda_2)$								
λ_1	**-5**	**-4.5**	**-4**	**-3.5**	**-3**	**-2.5**	**-2**	**-1.5**	**-1**
**1.025**	7.5% (0.649)	7.5% (0.636)	7.4% (0.641)	7% (0.647)	7.2% (0.574)	6.1% (0.576)	2.4% (0.901)	1.8% (0.46)	1.6% (0.167)
**1.05**	1.2% (0.444)	1.2% (0.465)	1.2% (0.426)	1.2% (0.433)	1.1% (0.412)	0.93% (0.363)	0.93% (0.206)	0.64% (0.135)	0.64% (0.0373)
**1.075**	0.55% (0.151)	0.55% (0.141)	0.57% (0.126)	0.57% (0.117)	0.57% (0.104)	0.57% (0.0917)	0.48% (0.0515)	0.48% (0.0301)	0% (n/a)
**1.1**	0.14% (0.115)	0.14% (0.085)	0.14% (0.081)	0.14% (0.0683)	0.14% (0.0763)	0.14% (0.046)	0.14% (0)	0% (n/a)	0% (n/a)
**1.125**	0.095% (0.0415)	0.095% (0.0175)	0.095% (0.018)	0.095% (0.0155)	0.12% (0.0096)	0.12% (0.0108)	0% (n/a)	0% (n/a)	0% (n/a)
**1.15**	0% (n/a)	0% (n/a)	0% (n/a)	0% (n/a)	0% (n/a)	0% (n/a)	0% (n/a)	0% (n/a)	0% (n/a)
**1.175**	0% (n/a)	0% (n/a)	0% (n/a)	0% (n/a)	0% (n/a)	0% (n/a)	0% (n/a)	0% (n/a)	0% (n/a)
**1.2**	0% (n/a)	0% (n/a)	0% (n/a)	0% (n/a)	0% (n/a)	0% (n/a)	0% (n/a)	0% (n/a)	0% (n/a)

***CDCA7L* rs4487645**

	$\log_{10}(\lambda_2)$								
λ_1	**-5**	**-4.5**	**-4**	**-3.5**	**-3**	**-2.5**	**-2**	**-1.5**	**-1**
**1.025**	18% (0.263)	18% (0.261)	18% (0.265)	18% (0.258)	17% (0.253)	17% (0.189)	16% (0.149)	15% (0.0655)	7.6% (0.0335)
**1.05**	7.8% (0.07)	7.7% (0.0679)	7.7% (0.0675)	7.6% (0.0696)	7.5% (0.0564)	6.8% (0.0469)	5.4% (0.0339)	4.2% (0.0142)	2.9% (0.0143)
**1.075**	3.8% (0.0165)	3.8% (0.0172)	3.8% (0.0152)	3.6% (0.0175)	3.5% (0.0138)	3.4% (0.0141)	3.4% (0.00495)	2.3% (0.0022)	2.3% (0.00337)
**1.1**	2.4% (0.00482)	2.4% (0.00401)	2.4% (0.00485)	2.4% (0.00348)	2.3% (0.00382)	2.3% (0.00138)	2.1% (0.0015)	2.2% (0.000613)	2.2% (0.000307)
**1.125**	1.7% (0.000961)	1.7% (0.00151)	1.6% (0.000515)	1.6% (0.000554)	1.6% (0.000416)	1.6% (0.000574)	1.6% (0.000158)	1.6% (0)	1.6% (0)
**1.15**	1.5% (0.000222)	1.5% (0.000178)	1.5% (0.000111)	1.5% (0)	1.5% (0.000378)	1.5% (0)	1.5% (0)	1.4% (0)	1.4% (0)
**1.175**	1.2% (0)	1.2% (2.7e-05)	1.2% (0.000135)	1.2% (0)	1.2% (0)	1.2% (0)	1.2% (0)	1.2% (0)	1.2% (0)
**1.2**	0.78% (0)	0.78% (0)	0.78% (0)	0.78% (0)	0.78% (0)	0.78% (0)	0.78% (0)	0.78% (0)	0.78% (0)

***CEP120* rs6595443**

	$\log_{10}(\lambda_2)$								
λ_1	**-5**	**-4.5**	**-4**	**-3.5**	**-3**	**-2.5**	**-2**	**-1.5**	**-1**
**1.025**	11% (0.456)	11% (0.45)	11% (0.448)	10% (0.408)	10% (0.415)	9.1% (0.368)	7.7% (0.295)	5.4% (0.154)	2.7% (0.0503)
**1.05**	3.8% (0.132)	3.8% (0.144)	3.8% (0.14)	3.8% (0.135)	3.7% (0.11)	3.4% (0.101)	3% (0.0566)	2.2% (0.0196)	1.9% (0.0103)
**1.075**	2.3% (0.0274)	2.3% (0.0297)	2.3% (0.0295)	2.2% (0.0267)	2.2% (0.025)	2.1% (0.0167)	2.1% (0.0127)	2.2% (0.00224)	1.9% (0.00152)
**1.1**	1.8% (0.00462)	1.8% (0.00539)	1.8% (0.00591)	1.8% (0.00518)	1.8% (0.00491)	1.8% (0.00218)	1.8% (0.00133)	2% (0.000111)	1.9% (0.00117)
**1.125**	1.6% (0.001)	1.6% (0.000723)	1.6% (0.00117)	1.6% (0.000877)	1.6% (0.000646)	1.6% (0.000492)	1.5% (0)	1.6% (0)	1.7% (0)
**1.15**	1.3% (9.26e-05)	1.3% (0.000148)	1.3% (0.000204)	1.3% (9.26e-05)	1.3% (7.41e-05)	1.3% (0)	1.3% (0.000111)	1.2% (0)	1% (0)
**1.175**	1% (0)	1% (0)	1% (2.38e-05)	0.99% (0)	0.99% (9.76e-05)	0.92% (0)	0.82% (0)	0.82% (0)	0.97% (0)
**1.2**	0.77% (0)	0.77% (0)	0.76% (0)	0.76% (0)	0.76% (0)	0.76% (3.17e-05)	0.76% (0)	0.76% (0)	0.64% (0)
