# Supplementary material for: Functional dissection of inherited non-coding variation influencing multiple myeloma risk
Source: Nat Commun. 2022 Jan 10;13:151. doi: 10.1038/s41467-021-27666-x (PMC8748989; doi:10.1038/s41467-021-27666-x)
Supplement: Supplementary file 3 — Description of Additional Supplementary Files [file 41467_2021_27666_MOESM3_ESM.pdf]

Description of Additional Supplementary Files

File Name: Supplementary Data 1

Description: Results of the two MPRA screens, sorted by absolute $\log_2$ score in L363 cells. The *P*- and *Q*-values are the final results from MPRA-score, integrating all barcodes that represent the variant (i.e., all three genomic windows and both strands).

File Name: Supplementary Data 2

Description: Motif analysis for rs78740585, rs2790444, rs3777182, rs3777183, rs11960493, rs6066832, rs62376437 using PERFECTOS-APE with these databases of motif models: (A) HOCOMOCO-11, (B) JASPAR, (C) HT-SELEX, (D) SwissRegulon, and (E) HOMER. The P-values are for motif-to-sequence alignment, as calculated by PERFECTOS-APE.
