# Supplementary material for: Functional dissection of inherited non-coding variation influencing multiple myeloma risk
Source: Nat Commun. 2022 Jan 10;13:151. doi: 10.1038/s41467-021-27666-x (PMC8748989; doi:10.1038/s41467-021-27666-x)
Supplement: Supplementary file 6 — Reporting Summary [file 41467_2021_27666_MOESM6_ESM.pdf]

nature research

Corresponding author(s): Nilsson

Last updated by author(s): Nov 4 2020

Reporting Summary

Nature Research wishes to improve the reproducibility of the work that we publish. This form provides structure for consistency and transparency in reporting. For further information on Nature Research policies, see our Editorial Policies and the Editorial Policy Checklist.

Statistics

For all statistical analyses, confirm that the following items are present in the figure legend, table legend, main text, or Methods section.

n/a	Confirmed	
[ ]	[x]	The exact sample size (n) for each experimental group/condition, given as a discrete number and unit of measurement
[ ]	[x]	A statement on whether measurements were taken from distinct samples or whether the same sample was measured repeatedly
[ ]	[x]	The statistical test(s) used AND whether they are one- or two-sided<br>*Only common tests should be described solely by name; describe more complex techniques in the Methods section.*
[x]	[ ]	A description of all covariates tested
[ ]	[x]	A description of any assumptions or corrections, such as tests of normality and adjustment for multiple comparisons
[x]	[ ]	A full description of the statistical parameters including central tendency (e.g. means) or other basic estimates (e.g. regression coefficient) AND variation (e.g. standard deviation) or associated estimates of uncertainty (e.g. confidence intervals)
[ ]	[x]	For null hypothesis testing, the test statistic (e.g. F, t, r) with confidence intervals, effect sizes, degrees of freedom and P value noted<br>*Give P values as exact values whenever suitable.*
[x]	[ ]	For Bayesian analysis, information on the choice of priors and Markov chain Monte Carlo settings
[x]	[ ]	For hierarchical and complex designs, identification of the appropriate level for tests and full reporting of outcomes
[ ]	[x]	Estimates of effect sizes (e.g. Cohen's d, Pearson's r), indicating how they were calculated

Our web collection on statistics for biologists contains articles on many of the points above.

Software and code

Policy information about availability of computer code

Data collection	PEAR v0.9.10, BWA-MEM v0.7.15, Trimmomatic v0.36, Bowtie2, SAMtools, Picard, BEDtools.
Data analysis	MATLAB 2020a, R v4.0.5, MPRAscore v1.0, g-Chromvar, caQTLseg v1.0 (available at: https://github.com/abhisheknrl/caQTLseg)

For manuscripts utilizing custom algorithms or software that are central to the research but not yet described in published literature, software must be made available to editors and reviewers. We strongly encourage code deposition in a community repository (e.g. GitHub). See the Nature Research guidelines for submitting code & software for further information.

Data

Policy information about availability of data

All manuscripts must include a data availability statement. This statement should provide the following information, where applicable:

- Accession codes, unique identifiers, or web links for publicly available datasets
- A list of figures that have associated raw data
- A description of any restrictions on data availability

The raw sequencing data for the MPRA experiment have been deposited in the Sequence Read Archive, accession no. PRJNA679966 (https://www.ncbi.nlm.nih.gov/bioproject/PRJNA679966). The ATAC-sequencing data have been deposited in the European Genome-phenome Archive (accession no. EGAS00001005394; https://ega-archive.org/studies/EGAS00001005394). Additionally, the following public data sets were used: Gene expression data for MM samples from the CoMMPASS study (https://themmrf.org/finding-a-cure/our-work/the-mmrf-commpass-study). Blood eQTL data from the eQTLgen consortium (eqtlgen.org); Gene expression data from the NCBI Gene Expression Omnibus (https://pubmed.ncbi.nlm.nih.gov/geo; accession numbers GSE111199, GSE24759, GSE15695, GSE4581, GSE19784, GSE26760, and GSE5900).

Field-specific reporting

Please select the one below that is the best fit for your research. If you are not sure, read the appropriate sections before making your selection.

- [x] Life sciences
- [ ] Behavioural & social sciences
- [ ] Ecological, evolutionary & environmental sciences

For a reference copy of the document with all sections, see nature.com/documents/nr-reporting-summary-flat.pdf

Life sciences study design

All studies must disclose on these points even when the disclosure is negative.

Sample size	The MPRA screen was done in two distinct cell lines, each assayed in three replicates.
Data exclusions	All three MPRA replicates for both cell lines were included in the analysis.
Replication	Effects identified by MPRA were validated using luciferase assays for 20 variants. In these experiments, all variants except one showed effects in the same direction as in the MPRA.
Randomization	Not applicable. An MPRA screen is an in vitro assay that does not involve random assignment of subjects to test groups.
Blinding	Not applicable. An MPRA screen is an in vitro assay that does not require blinding.

Reporting for specific materials, systems and methods

We require information from authors about some types of materials, experimental systems and methods used in many studies. Here, indicate whether each material, system or method listed is relevant to your study. If you are not sure if a list item applies to your research, read the appropriate section before selecting a response.

Materials & experimental systems

n/a	Involved in the study	
☐	☒	Antibodies
☐	☒	Eukaryotic cell lines
☒	☐	Palaeontology and archaeology
☒	☐	Animals and other organisms
☐	☒	Human research participants
☒	☐	Clinical data
☒	☐	Dual use research of concern

Methods

n/a	Involved in the study	
☒	☐	ChIP-seq
☒	☐	Flow cytometry
☒	☐	MRI-based neuroimaging

Antibodies

Antibodies used	We used antibodies against POU2F1 (cat no. sc-8024; Santa Cruz) and IRF4 (cat no. 646412; Biolegend).
Validation	According to manufacturers, sc-8024 has been validated by ectopic over-expression of POU2F1 and 646412 has been validated by Chromatin-Immunoprecipitation and Western blot assays in HeLa and Ramos cells.

Eukaryotic cell lines

Policy information about cell lines

Cell line source(s)	L363, MOLP8 and OPM2, obtained from DSMZ (accession numbers ACC49 , ACC569 and ACC50 , respectively).
Authentication	The cell lines were genotyped for the CRISPR-edited/deleted SNPs, and found to the expected genotype, as compared to publicly available genotype data in the Cancer Cell Line Encyclopedia.
Mycoplasma contamination	The cell lines were not tested for mycoplasma.
Commonly misidentified lines (See ICLAC register)	No commonly misidentified cell lines were used in the study.

Human research participants

Policy information about studies involving human research participants

Population characteristics	For ATAC-seq, we used plasma cells from MM patients, drawn at diagnosis. Clinical information was not available.
Recruitment	The ATAC-seq samples were dervied from the Norwegian MM biobank, which collects samples from newly diagnosed MM patients from all of Norway, without additional inclusion/exclusion criteria.
Ethics oversight	Ethics approval numbers REK 2014/97 (Norway) and 2019-06386 (Sweden).

Note that full information on the approval of the study protocol must also be provided in the manuscript.
